# Genome-wide CRISPR-screening identifies p53 as regulator of cancer cell sensitivity to the histone evicting anthracycline aclarubicin

**DOI:** 10.1038/s41419-026-09138-6

**Published:** 2026-08-05

**Authors:** Sabina Y. van der Zanden, Koen Schipper, Merle A. van Gelder, Amina Teunisse, Jay Sarthy, Aart G. Jochemsen, Ruud H. M. Wijdeven, Jacques Neefjes

**Affiliations:** 1 Department of Cell and Chemical Biology, ONCODE institute, Leiden University Medical Center, Leiden, The Netherlands; 2 Ben Towne Center, Seattle Children’s Research Institute, Seattle, Washington and Department of Pediatrics, University of Washington, Seattle, WA USA; 3 Alzheimer Center Amsterdam, department of Neurology, Amsterdam Neuroscience Amsterdam, University Medical Center Amsterdam, Amsterdam, The Netherlands

**Keywords:** Chemotherapy, Tumour-suppressor proteins

## Abstract

The anthracycline family, with its prime member doxorubicin, is one of the cornerstones in cancer chemotherapy and acts by poisoning topoisomerase II, resulting in DNA double-stranded breaks. One of its members, aclarubicin, is considered a distinct member of this family due to its inability to inflict DNA double-strand breaks. Despite this, aclarubicin is an effective anti-cancer drug by evicting histones, resulting in chromatin damage. How aclarubicin-induced chromatin damage induces cell death remains largely unknown. Here, we performed a genome-wide CRISPR screen to identify factors regulating sensitivity of cells to aclarubicin and identified p53 as a critical factor for cellular sensitivity. Even though aclarubicin does not induce DNA breaks, treatment resulted in swift activation of ATM independent of the MRN complex, which stabilized and activated p53, resulting in apoptosis and cell cycle arrest. Furthermore, response to aclarubicin treatment could reasonably be predicted by the p53-status of cell lines. These data suggest that ATM and p53 can be activated for apoptosis induction by at least two different pathways, DNA- and chromatin damage. Together, these data suggest p53 could be an important factor to stratify patients for treatment with Aclarubicin.

## Introduction

Aclarubicin belongs to the family of anthracycline drugs, which are among the most widely used anti-cancer drugs. This family, including doxorubicin, epirubicin, daunorubicin and idarubicin, is used to treat a wide spectrum of tumors [1, 2]. The classical mechanism of action of anthracyclines is poisoning of topoisomerase II following intercalation of these compounds into DNA and the formation of topoisomerase II-DNA complexes [3, 4]. The resulting DNA double-strand breaks activate p53 and DNA damage response pathways for cell cycle arrest and/or cell death [5]. Aclarubicin is a unique member of the anthracycline family. It inhibits topoisomerase at a different step in the catalytic cycle, preventing enzyme binding to the DNA [3, 4]. As a consequence, treatment with aclarubicin does not lead to induction of DNA breaks [6–8]. The anthracyclines differ from other topoisomerase II poisons, such as the podophyllotoxin etoposide, in that they have a second mechanism of action. When the anthraquinone moiety of anthracyclines intercalates into the DNA double helix, the aminosugar moiety emanates into the DNA minor groove, causing destabilization of nucleosomes and eviction of histones at defined areas in the genome [4, 6, 9]. This activity has multiple consequences, including attenuated DNA damage responses, altered transcriptomics, and deregulation of the epigenome at defined regions of the genome, collectively referred to as chromatin damage [6, 7]. Evaluation of this activity amongst various anthracycline variants has shown that chromatin damage is essential to the effectiveness of this class of anti-cancer drugs [8, 10, 11]. This is illustrated by the anthracycline amrubicin and the topoisomerase inhibitor etoposide, which are abstained from chromatin damage activity and are considerably less cytotoxic [8]. Furthermore, the combination of DNA- and chromatin damage (as is the case for doxorubicin, epirubicin, daunorubicin and idarubicin) has been shown to conspire to cause a number of severe side effects [8], including dose-dependent cardiotoxicity, therapy-related malignancies and gonadotoxicity [12, 13]. On the contrary, variants deficient for DNA damage activity, such as aclarubicin, lack these severe side effects displayed by the other family members [8].

Although aclarubicin is an effective anti-cancer drug used in the treatment of AML patients [14], the mechanism by which chromatin damage leads to cell death is poorly understood. Therefore, we performed a genome-wide CRISPR knockout screen to identify regulators of aclarubicin sensitivity, yielding p53 as the most prominent hit. Indeed, p53 depletion decreased sensitivity to aclarubicin, and mutational status of p53 could be used as a proxy for sensitivity of cells to aclarubicin. Mechanistically, aclarubicin treatment leads to the phosphorylation of ATM, leading to DNA-binding, phosphorylation, and stabilization of p53, resulting in induction of apoptosis even in the absence of DNA breaks and activation of the MRN complex. Thus, chromatin damage induced by aclarubicin can be sensed by p53 and translated into a cellular response also observed in a DNA damage response, leading to the induction of cell death. In the absence of p53, higher aclarubicin concentrations still induce cell death. Together, this uncovers a second mechanism of induction of p53-mediated apoptosis by anthracycline cancer drugs.

## Results

### CRISPR-screening identifies p53 as regulator of aclarubicin sensitivity

**Fig. 1:**
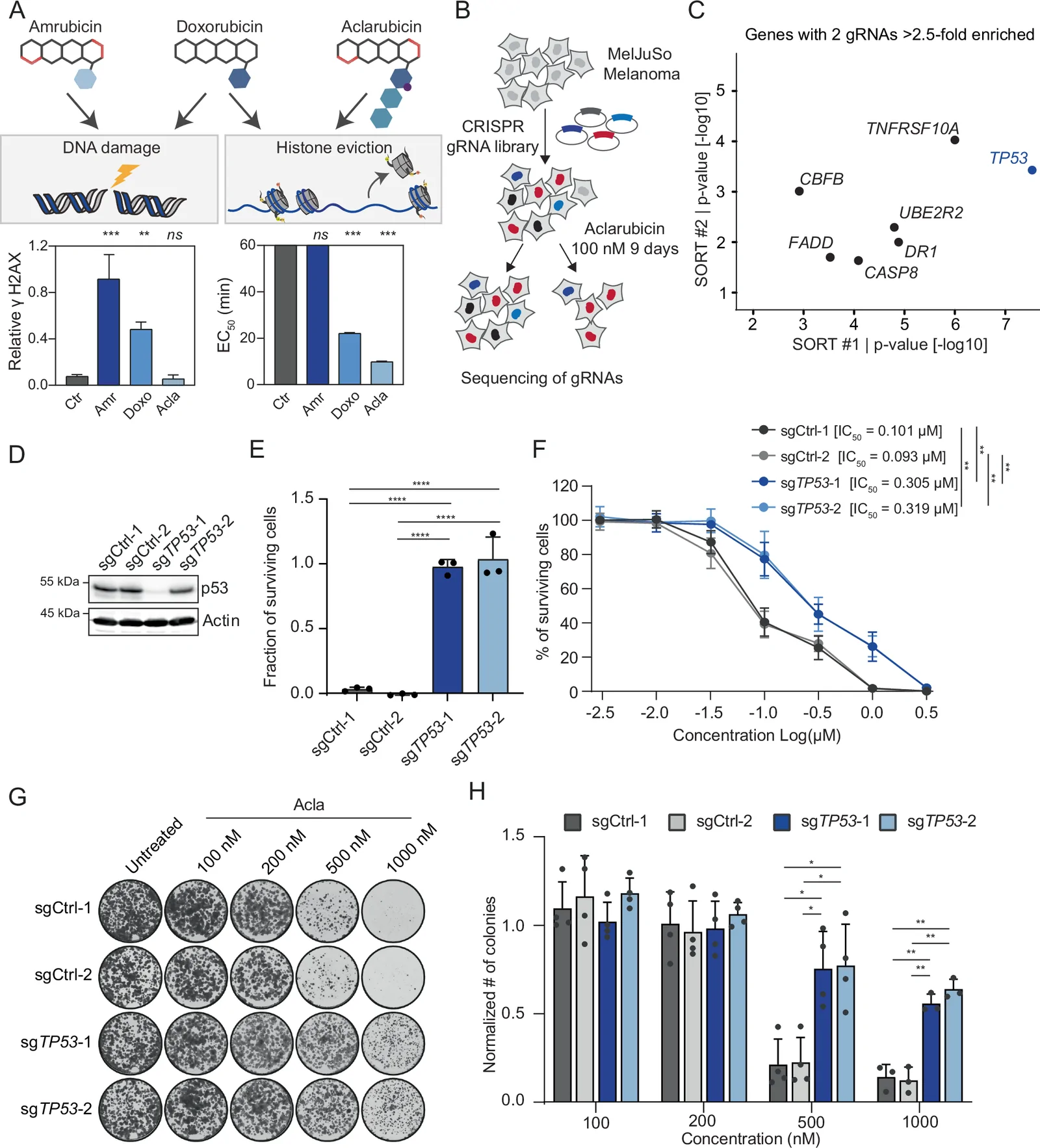
genome-wide CRISPR screening identifies p53 as a regulator of sensitivity to Aclarubicin. **A** Top panel: Schematic overview of the DNA damage (via targeting topoisomerase II) and chromatin damage (via eviction of histones) activity of amrubicin, doxorubicin, and aclarubicin. Bottom panel: DNA double-strand breaks induced by these three anthracyclines upon treatment of 10 µM for 2 hours in MelJuSo cells and histone eviction capacity of these drugs indicated by EC_50_ (time in which 50% of the histones are evicted). Ordinary one-way ANOVA with multiple comparisons, ** *p* < 0.01, *** *p* < 0.001, **** *p* < 0.0001. **B** Schematic representation of the screening outline. MelJuSo melanoma cells were transduced with the Brunello genome-wide CRISPR knockout library, and cells were either or not treated with aclarubicin (100 nM) for 9 days, followed by gRNA sequencing. **C** Genes for which at least two different gRNAs were enriched (> 2.5-fold) in both aclarubicin-treated samples and which were significantly enriched based on RSA analysis. **D** MelJuSo cells transduced with the indicated CRISPR constructs were analyzed for p53-expression by Western blot. **E** MelJuSo cells as in (**C**) were treated with HMD201 for 3 days, and viability was assessed and normalized to untreated cells. *n* = 3 independent experiments. Ordinary one-way ANOVA with multiple comparisons, **** *p* < 0.0001. **F** Indicated MelJuSo cells were treated for 2 hours with various concentrations of aclarubicin, after which the drug was washed out, and cells were left to grow for another 3 days before assessing cell viability. *n* = 3 independent experiments. IC_50_ for each cell line is indicated. Two-tailed *t*-test, ** *p* < 0.01. **G** Colony formation assay for indicated MelJuSo cells. Cells were treated for 2 hours with aclarubicin, after which the drug was removed by washing and cells were left to grow out for 6–9 days before fixation and staining with Crystal Violet. **H** Quantification of four independent colony formation experiments; signal was normalized to the respective untreated cells. Ordinary two-way ANOVA with multiple comparisons, * *p* < 0.05, ** *p* < 0.01.

Doxorubicin is a widely used chemotherapeutic that by intercalation into the DNA traps the enzyme topoisomerase II, forming a drug-DNA-enzyme adduct resulting in the formation of DNA double-strand breaks by preventing the religation step [3]. Furthermore, drug intercalation into the DNA induces chromatin damage via eviction of histones (Fig. 1A) [6, 15]. Unlike doxo-, epi-, dauno- and idarubicin, this dual action mechanism is not observed for all clinical relevant anthracyclines drugs, where amrubicin only induces DNA double-strand breaks (Fig. 1A and Fig. S1A), and aclarubicin has only histone eviction activity (Fig. 1A and Fig. S1B). However, how chromatin damage activity induces cell death remains unclear. To identify proteins contributing to aclarubicin-induced cell death following chromatin damage, we performed a genome-wide CRISPR knockout screen. MelJuSo melanoma cells were used as a model cell line, since these are sensitive to aclarubicin (IC_50_ of about 100 nM) and take up the drug efficiently [6, 8]. Cells were transduced with the Brunello genome-wide CRISPR library (4 gRNAs per gene) and treated five days later with aclarubicin for nine days at a concentration of 100 nM, a sublethal concentration (Fig. 1B). After this, surviving cells were harvested, genomic DNA was isolated, sequenced and compared to the untreated control. Hit calling was based on statistical significance in both biological replicates using RSA analysis, as well as an absolute fold enrichment of > 2.5 for at least 2 gRNAs per gene. This yielded two prominent hits: apoptotic TRAIL receptor DR4 (encoded by *TNFRSF10A*) and tumor suppressor p53 (*TP53*) (Fig. 1C and Fig. S1C). Less prominent hits included Caspase-8 and FADD, the two signaling adaptors for DR4, as well as the transcriptional regulators DR1 and CBFB. However, the fold enrichments for these hits were low, suggesting only a minor contribution of these genes to aclarubicin resistance. To identify potential additional factors responsible for aclarubicin sensitivity, we repeated the screen in a p53-deficient background and used a higher dosage of aclarubicin (250 nM) to account for the decreased sensitivity of p53-deficient cells (Fig. 1F). However, this yielded no additional candidate resistance factors (Fig. S2 and Supplementary Table 1). Overall, these results suggest a role for p53 and apoptotic signaling in aclarubicin sensitivity.

To validate the involvement of p53 in aclarubicin sensitivity, we transduced MelJuSo cells with the top two gRNAs from the screen, creating two different pools of p53-deficient cells. One of these yielded a robust depletion of p53, whereas gRNA #2 (which targets the domain of *TP53* involved in DNA binding) resulted in a mutated p53 protein (Fig. 1D and S1D). Importantly, both cell lines were completely insensitive to treatment with the MDM2-inhibitor RG7112 that stabilizes p53, validating the generation of two functional *TP53*-knockout cell lines (Fig. 1E). These p53-deficient MelJuSo cells were more resistant to aclarubicin following either a short pulse treatment or continuous 72 hours exposure (Fig. 1F and Fig. S1E), as well as in long-term colony-forming assays (Fig. 1G, H). Thus, our CRISPR-screen identified a role for p53 in sensitivity of cells to aclarubicin.

### p53 is a major determinant in cellular sensitivity to aclarubicin

**Fig. 2:**
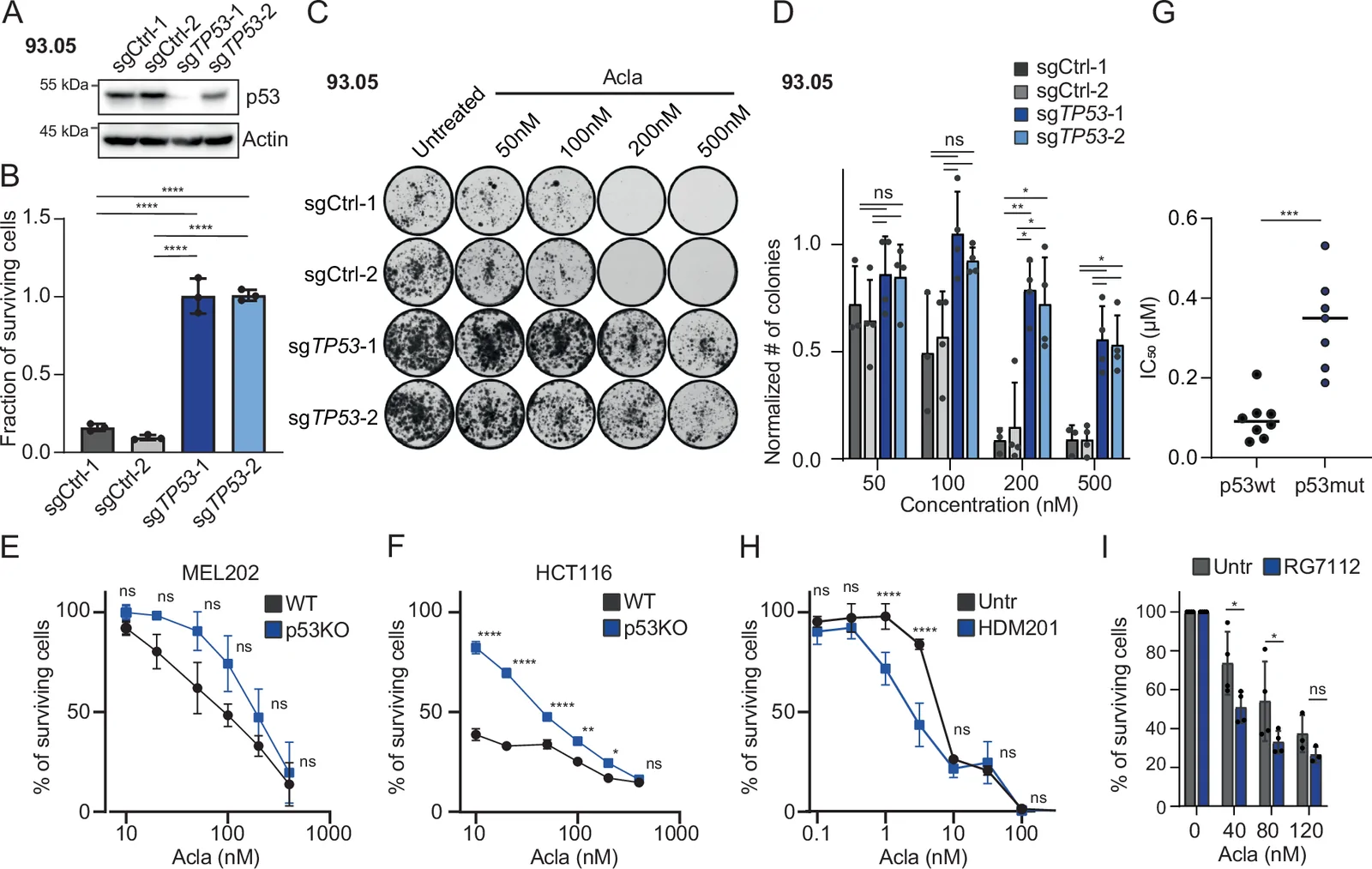
p53 is a major determinant in the sensitivity of cells to aclarubicin. **A** 93.05 cells transduced with the indicated CRISPR constructs were analyzed for p53-expression using Western blot. **B** 93.05 cells as in (**A**) were treated with HMD201 for 3 days, and viability was assessed and normalized to untreated cells. *n* = 3 independent experiments. Ordinary one-way ANOVA with multiple comparisons, ****p < 0.0001. **C** Colony-forming assay for 93.05 melanoma cells transduced with the indicated CRISPR constructs. Cells were treated with aclarubicin for 2 hours, washed, and 7–9 days later fixed and stained with Crystal Violet. **D** Quantification of three independent experiments; signal was normalized to the respective untreated cells. Ordinary two-way ANOVA with multiple comparisons, * *p* < 0.05, ** *p* < 0.01, ns = not significant. **E** Mel202 and **F** HCT116 sgCtrl cells or sg*TP53* were treated with aclarubicin for 2 hours, washed, and 3 days later viability was assessed. Mel202 data represent a biological replicate, HCT116 data represent a technical triplicate. Ordinary two-way ANOVA with multiple comparisons, * *p* < 0.05, ** *p* < 0.01, **** *p* < 0.0001. **G** The IC_50_ was determined from 15 cell lines from different tissues (8 p53 WT and 7 p53 mutant) by exposing cells to aclarubicin for 2 hours and measuring viability three days later. IC_50_ represents the average of three independent experiments, and cell lines were clustered based on p53 status. Two-tailed *t*-test, *** *p* < 0.001. **H** MelJuSo cells were treated with the indicated concentrations of aclarubicin for 72 hours, and 500 nM HDM201 was added when indicated. Three days later, viability was measured and normalized to the untreated control, either treated or not treated with HDM201. Data represent four independent experiments. Ordinary two-way ANOVA with multiple comparisons **** *p* < 0.0001. **I** MEL202 cells were treated for 2 hours with the indicated concentrations of aclarubicin, after which the aclarubicin was washed away, and cells were left to grow out in the presence or absence of 250 nM RG7112. Three days later, cell viability was measured and normalized to the non-aclarubicin-treated controls. Data represent three (120 nM) or four (40 and 80 nM) independent experiments. Statistical significance was determined by Ordinary two-way ANOVA with multiple comparisons *p < 0.05.

To test whether the function of p53 is conserved across multiple cell lines, we used the same approach to deplete p53 from 93.05 melanoma cells (Fig. 2A, B and S3A, B). In both long-term and short-term growth assays, p53-deficient cells were more resistant to aclarubicin (Fig. 2C, D and Fig. S3C). Similarly, p53-deficient MEL202 uveal melanoma (Fig. S3D, E) and HCT116 colon cancer lines were less sensitive to aclarubicin (Fig. 2E, F). Finally, the untransformed RPE1 cells were also less sensitive to aclarubicin when p53 was depleted (Fig. S3F–H). To verify whether p53-status as a critical determinant for the sensitivity of cells to aclarubicin, we tested a panel of 15 different p53-wt and p53-mutant cell lines. Interestingly, based on the IC_50_-values, the two groups almost completely segregated, arguing that the sensitivity of cells to aclarubicin can be predicted by their p53-mutational status (Fig. 2G and Supplementary Table 2). This dependence on p53 activity suggests that aclarubicin might synergize with p53-stabilizing drugs. To test this, MelJuSo cells were incubated with a low dose of HDM201 (p53-MDM2 inhibitor) in the presence of different doses of aclarubicin. While aclarubicin single treatment already decreased cell viability, the combination treatment was even more effective, indicating that p53 stabilization by HDM201 acts synergistically with aclarubicin-induced chromatin damage activity (Fig. 2H). A similar observation was made for MEL202 cells, where co-treatment of the MDM2 antagonist RG7112 synergized with various concentrations of aclarubicin (Fig. 2I). These data further support the observation that p53 status is a major determinant for sensitivity towards the anthracycline drug aclarubicin.

### Aclarubicin stabilizes and activates p53

**Fig. 3:**
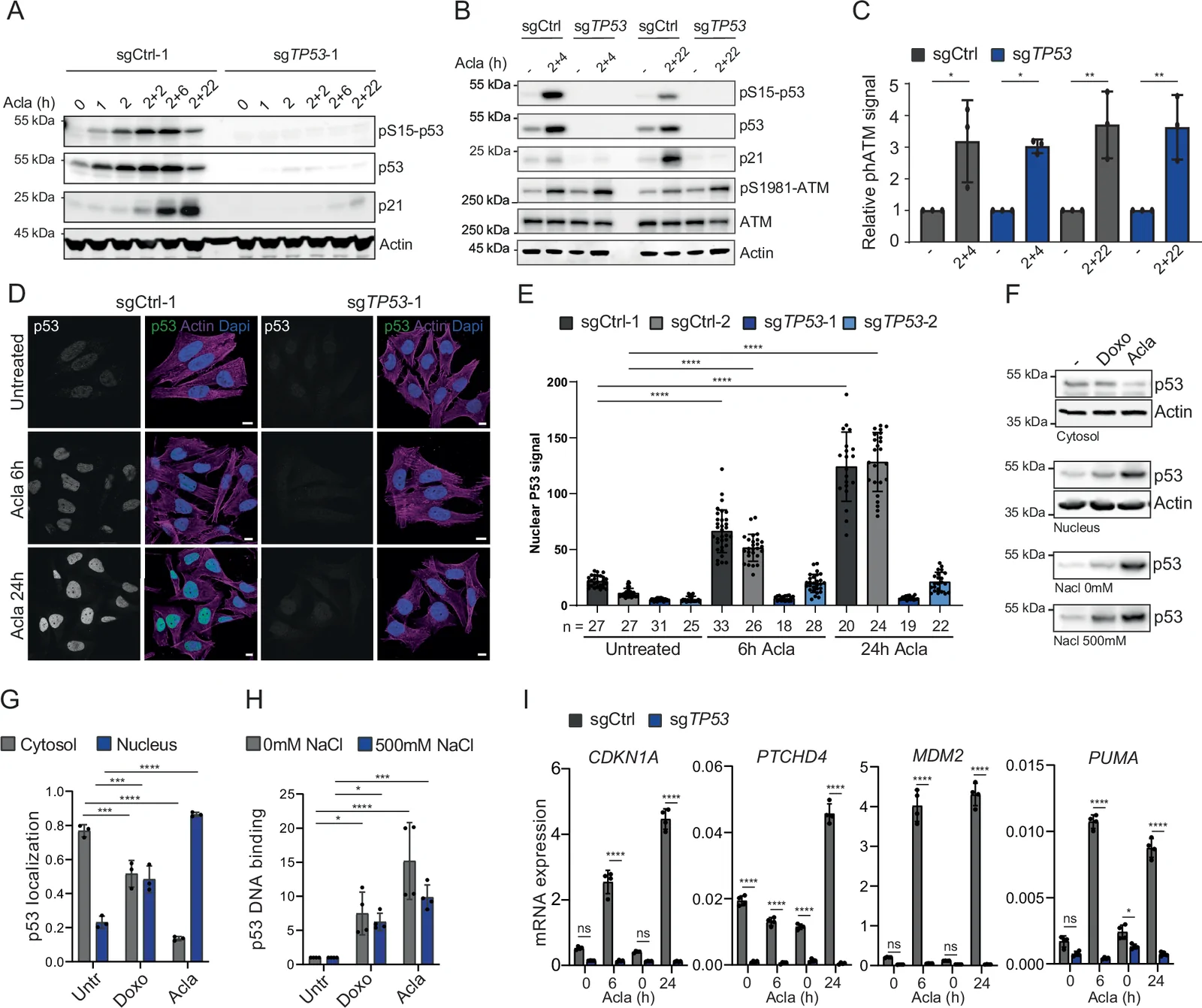
Aclarubicin induces phosphorylation and activation of p53. **A** MelJuSo cells were exposed to 200 nM aclarubicin for the indicated time points and washed away after 2 hours, after which cells were left to recover for the indicated time points before lysis and analysis by Western blot. Representative image of 4 independent experiments. **B** 93.05 melanoma cells were exposed to aclarubicin for 2 hours, washed and lysed 4 hours or 22 hours later, before analysis of the indicated proteins by Western blot. **C** Quantification of relative phATM signal of *n* = 3 independent experiments. Ordinary one-way ANOVA with multiple comparisons, * *p* < 0.05, ** *p* < 0.01. **D** Representative confocal image of fixed MelJuSo cells stained for endogenous p53. Cells were treated with 200 nM aclarubicin for 2 hours, washed, and fixed at the indicated time points post-treatment. DAPI and actin are stained as nuclear and cytosolic markers, respectively. Scale bar; 10 µm. **E** Quantification of endogenous p53 levels. *n* = number of cells a*n*alyzed per condition. Ordinary two-way ANOVA with multiple comparisons, **** *p* < 0.0001. **F** Chromatin binding assay for endogenous p53 in MelJuSo cells. Cells were treated with 10 µM doxo- or aclarubicin for 45 minutes followed by fractionation. P53 localization and DNA binding were analyzed by Western Blot. **G** Quantification of cytosolic and nuclear p53 localization. Ordinary two-way ANOVA with multiple comparisons *** *p* < 0.001, **** *p* < 0.0001. **H** Quantification of DNA binding. *n* = 4 independent experiments. Ordinary two-way ANOVA with multiple comparisons * *p* < 0.05, *** *p* < 0.001, **** *p* < 0.0001. **I** 93.05 cells were exposed for 2 h to 200 nM aclarubicin, and RNA was isolated 4 hours (6 hours) or 22 hours (24 hours) post-treatment. Expression of indicated mRNA was detected by qRT-PCR and normalized to housekeeping gene expression. *n* = 2 independent experiments; data represent technical quadruplicates. Two-way ANOVA with multiple comparisons, * *p* < 0.05, ** *p* < 0.01, *** *p* < 0.001.

The fact that aclarubicin induces histone eviction [6] together with the involvement of p53 in aclarubicin-induced cytotoxicity suggests that p53 can sense the inflicted chromatin damage. p53-activating signals, such as DNA damage, lead to p53 phosphorylation and stabilization, after which p53 induces the expression of target genes involved in cell cycle arrest and cell death [16]. To study the function of p53 upon induction of chromatin damage, we tested whether p53 levels were affected by aclarubicin exposure. Already after 2 hours of treatment, p53 stabilization and enhanced p53-S15 phosphorylation was observed by Western blot (Fig. 3A). In addition, aclarubicin treatment resulted in a p53-dependent increased expression of p53-target genes such as p21 (Fig. 3A, B and Fig. S4A, B), MDM2 and PUMA (Fig. S4B). For other genotoxic triggers, ATM kinase is activated by phosphorylation, subsequently inducing p53 phosphorylation [17]. ATM was also phosphorylated following aclarubicin treatment, as well as KAP1, another ATM substrate, suggesting that the histone-evicting anthracycline aclarubicin likewise activates ATM and the subsequent stress response (Fig. 3B, C). Furthermore, nuclear stabilization of endogenous p53 by aclarubicin treatment was observed by microscopy (Fig. 3D, E and Fig. S4C). Interestingly, the activation of ATM was independent of the MRN complex, as knockout of MRE11 or NBS1 (encoded by *MRE11* and *NBN*, respectively) in MelJuSo cells did not prevent ATM activation or subsequent p53 phosphorylation and stabilization after aclarubicin exposure (Fig. S4D, E).

To confirm that enhanced nuclear p53 is also associated with DNA to drive transcription, we analyzed DNA-binding of p53 using a chromatin-association assay. Indeed, p53 was found to accumulate in the nucleus upon treatment with aclarubicin (doxorubicin was used as a positive control [18]), while cytosolic p53 levels were reduced (Fig. 3F, G). Furthermore, aclarubicin treatment resulted in more p53 binding to DNA (Fig. 3F, H). To verify that aclarubicin treatment activates p53, subsequently leading to DNA binding and transcription of its target genes, we performed qRT-PCR experiments for different p53-target genes. Expression of several p53-target genes was strongly increased by aclarubicin, whereas expression of these genes was unaltered in p53-null cells upon aclarubicin treatment (Fig. 3I, S4F, G). Taken together, these data indicate that anthracycline variants acting only by histone eviction activity, such as aclarubicin, also activate p53, which results in increased p53 target gene expression.

### Aclarubicin activates p53 to induce apoptosis

**Fig. 4:**
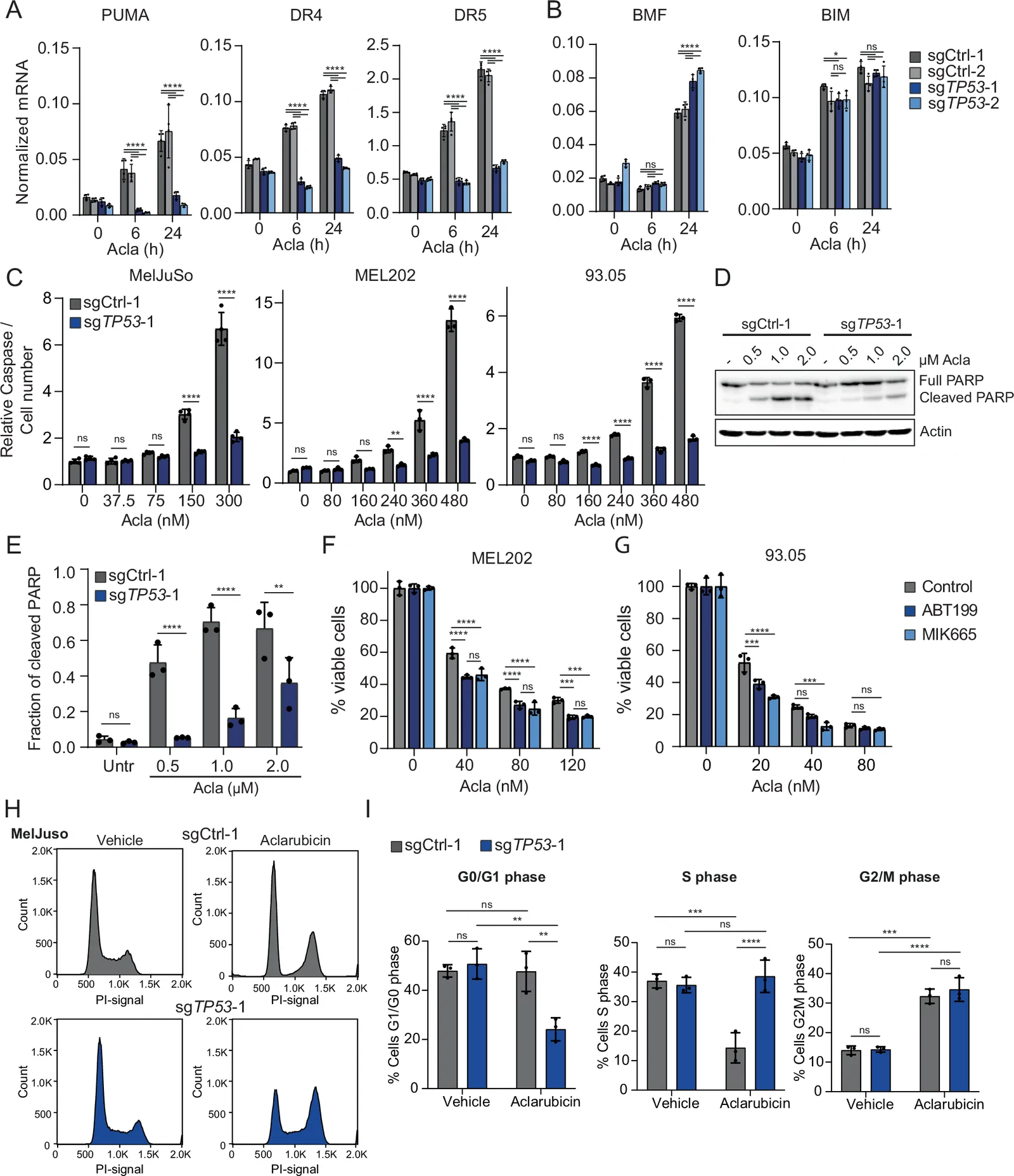
Aclarubicin induces p53-dependent apoptosis and cell cycle arrest. **A**, **B** Indicated MelJuSo cells were treated with 200 nM aclarubicin for 2 hours and RNA was isolated 4 hours (6 hours) or 22 hours (24 hours) post-treatment. Expression of indicated mRNA was detected by qRT-PCR and normalized to housekeeping gene expression. *n* = 2 independent experiments; data represent technical triplicates. Statistical significance was determined by a Two-way ANOVA with multiple comparisons; ns is not significant, *p < 0.05, ** *p* < 0.01. **C** Indicated cells were exposed to different concentrations of aclarubicin for 2 hours. Three days later, cell viability and Caspase-Glo signal were detected. Caspase-Glo signal was normalized to the cell count. *n* = 2 independent experiments; data represent technical triplicates. Two-way ANOVA with multiple comparisons, ** *p* < 0.01, **** *p* < 0.0001. **D** Indicated MelJuSo cells were exposed to different concentrations of aclarubicin for 2 h, 24 h post treatment cells were collected. PARP cleavage was analyzed by Western blot. Position of PARP and its cleaved form is indicated. Actin was used as a loading control. **E** Quantification of the fraction of cleaved PARP. Two-way ANOVA with multiple comparisons, ns is not significant, ** *p* < 0.01, **** *p* < 0.0001. **F**, **G** Indicated cell lines were treated for 2 hours with different concentrations of aclarubicin; the drug was washed away, and cells were left to grow out in the presence or absence of 200 nM MIK665 or 4 µM ABT-199. Three days later, cell viability was measured and normalized to the non-aclarubicin-treated controls. Data represent three independent experiments. Two-way ANOVA with multiple comparisons, *** *p* < 0.001, **** *p* < 0.0001. Representative histograms (**H**) and quantifications (**I**) of propidium iodide staining of MelJuSo sgCtrl-1 and sg*TP53*-1 cells exposed to 200 nM aclarubicin for 2 hours and collected 22 hours later. Data represent three independent experiments. Two-way ANOVA with multiple comparisons, **p < 0.01 ***p < 0.001, ****p < 0.0001.

p53 induces the expression of several pro-apoptotic genes [16], suggesting that aclarubicin-induced p53 activation inflicts cell death via apoptosis. In line with this, several pro-apoptotic factors such as PUMA, DR4 and DR5 were upregulated by aclarubicin-treatment (Figs. 3I, 4A), whereas the anti-apoptotic factor survivin was downregulated (Fig. S5A, B). Most of this effect was governed by p53, since it was absent in the p53-null cells. However, for some factors, such as the pro-apoptotic factors BIM and BMF, increased expression was also observed in the p53-deficient cells (Fig. 4B). Furthermore, direct apoptosis induction by aclarubicin was measured using a Caspase-GLO assay. In all three melanoma cell lines tested, a robust dose-dependent increase in caspase activity after aclarubicin was observed, which was largely absent in cells deficient for p53 (Fig. 4C). Similar results were obtained when detecting the cleavage of caspase substrate PARP by Western blot (Fig. 4D, E). Several activators of apoptosis have been developed, such as inhibitors of the anti-apoptotic factors BCL-2 (ABT-199, also known as venetoclax) and MCL-1 (MIK665) [19]. We tested whether these inhibitors synergize with aclarubicin treatment to increase cell toxicity. When using sublethal concentrations of either ABT-199 or MIK665, aclarubicin synergized with these inhibitors to tip the balance and induce cell death in both MEL202 and 93.05 cells (Fig. 4F, G).

The induction of p21 by aclarubicin suggests that some cells might undergo cell cycle arrest. Therefore, we performed a cell cycle analysis on p53-proficient and -deficient cells by flow cytometry (Fig. 4H, I and Fig. S6A–D). In p53-proficient cells, aclarubicin drastically reduces the amount of cells in S-phase, coinciding with an increase in the amount of cells in G2/M, suggesting cell cycle arrest in both the G1 and G2 phases. In p53-deficient cells, aclarubicin does not affect the number of cells in S-phase but increases the number of cells in G2/M at the cost of a reduced number of cells in G1/0. In addition, we observed a p53-dependent decrease in the expression of the E2F1 transcription factor and MAD2L1 after aclarubicin exposure (Fig. S5 A–C). Together, these data suggest that aclarubicin induces both apoptosis and cell cycle arrest that involves p53-dependent induction of pro-apoptotic genes and induction of p21.

### ATM mediates aclarubicin-induced activation of p53

**Fig. 5:**
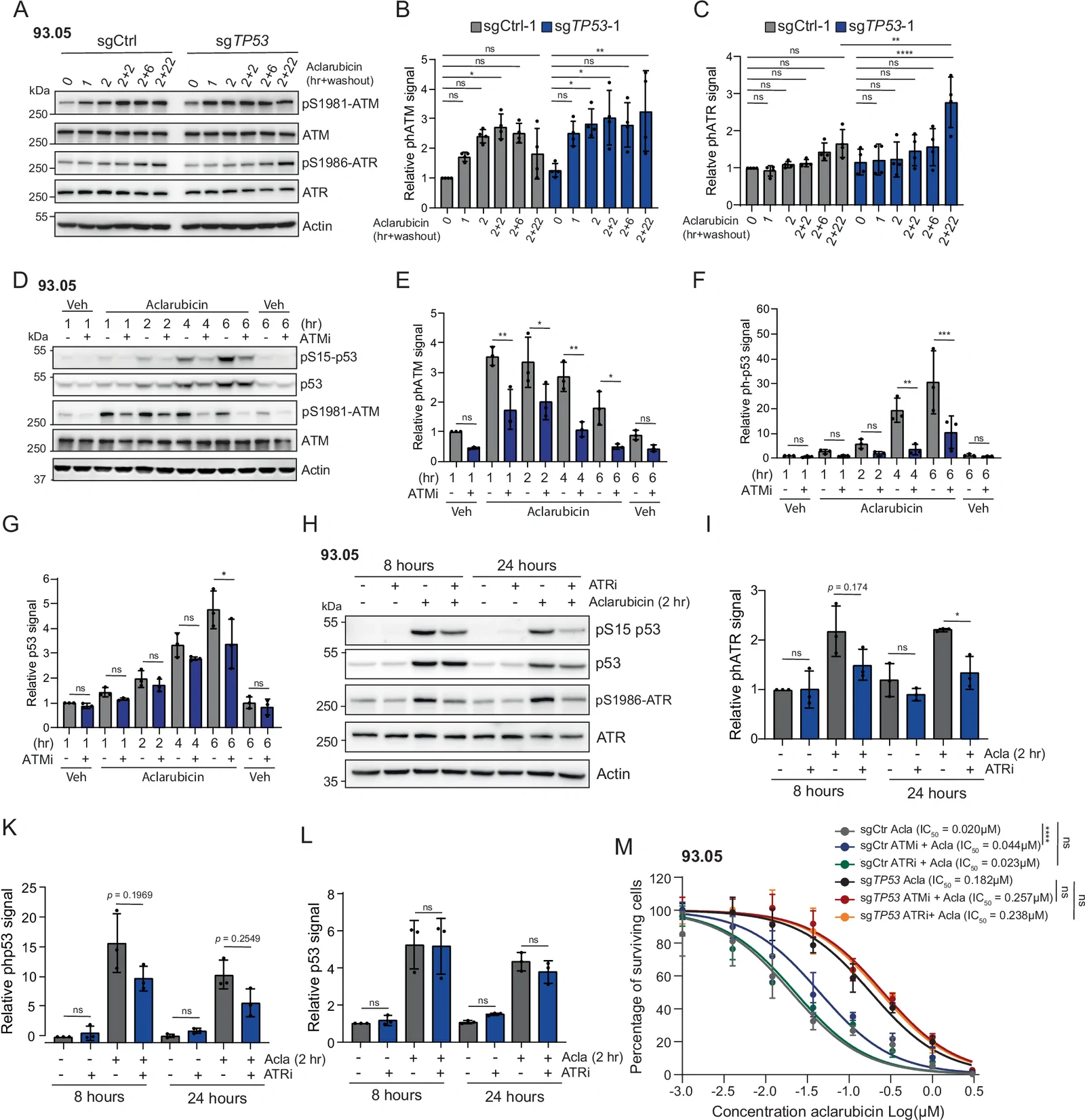
ATM but not ATR activation drives p53-mediated apoptosis after aclarubicin exposure. **A** 93.05 p53- proficient and -deficient melanoma cells were exposed to aclarubicin for 2 hours, washed, and lysed 2 hours, 6 hours or 22 hours later, followed by analysis of the indicated proteins by Western blot. **B**, **C** Quantification of relative pS1981-ATM and pT1989-ATR signal. *n* = 4 independent experiments. Ordinary one-way ANOVA with multiple comparisons, *p < 0.05, **p < 0.01, ****p < 0.0001. **D** 93.05 cells were pretreated with the ATM inhibitor KU-55933 or vehicle (VEH) for 1 hour prior to 200 nm aclarubicin/Vehicle exposure for 1, 2, 4, or 6 hours, followed by cell lysis and analysis of the indicated proteins by Western blot. Quantification of relative pS15-p53 (**E**), p53 (**F**), and pS1981-ATM (**G**) signal of *n* = 3 independent experiments. Ordinary one-way ANOVA with multiple comparisons, * *p* < 0.05, ** *p* < 0.01, *** *p* < 0.001, **** *p* < 0.0001. **H** 93.05 cells were pretreated with the ATR inhibitor VE821 or vehicle (VEH) for 1 hour prior to 200 nM aclarubicin/vehicle/VE-821 exposure for 2, followed by washout and another 6 or 22 hours of recovery in the presence of VE-821/Vehicle followed by cell lysis and analysis of the indicated proteins by Western blot. **I-L** Quantification of relative pS15-p53 (**I**), p53 (**K**), and pT1989-ATR (**L**) signal of *n* = 3 independent experiments. Ordinary one-way ANOVA with multiple compariso*n*s, * *p* < 0.05, ** *p* < 0.01, *** *p* < 0.001, **** *p* < 0.0001. **M** 93.05 p53-proficient and -deficient melanoma cells were exposed to different concentrations of aclarubicin in the presence or absence of 10 µM ATM inhibitor KU-55933 (KU) or 5 µM ATR inhibitor VE-821 (VE) for 3 days, followed by cell viability analysis. The signal was normalized to the non-aclarubicin-treated controls. Ordinary one-way ANOVA with multiple comparisons, **** *p* < 0.0001.

**Fig. 6:**
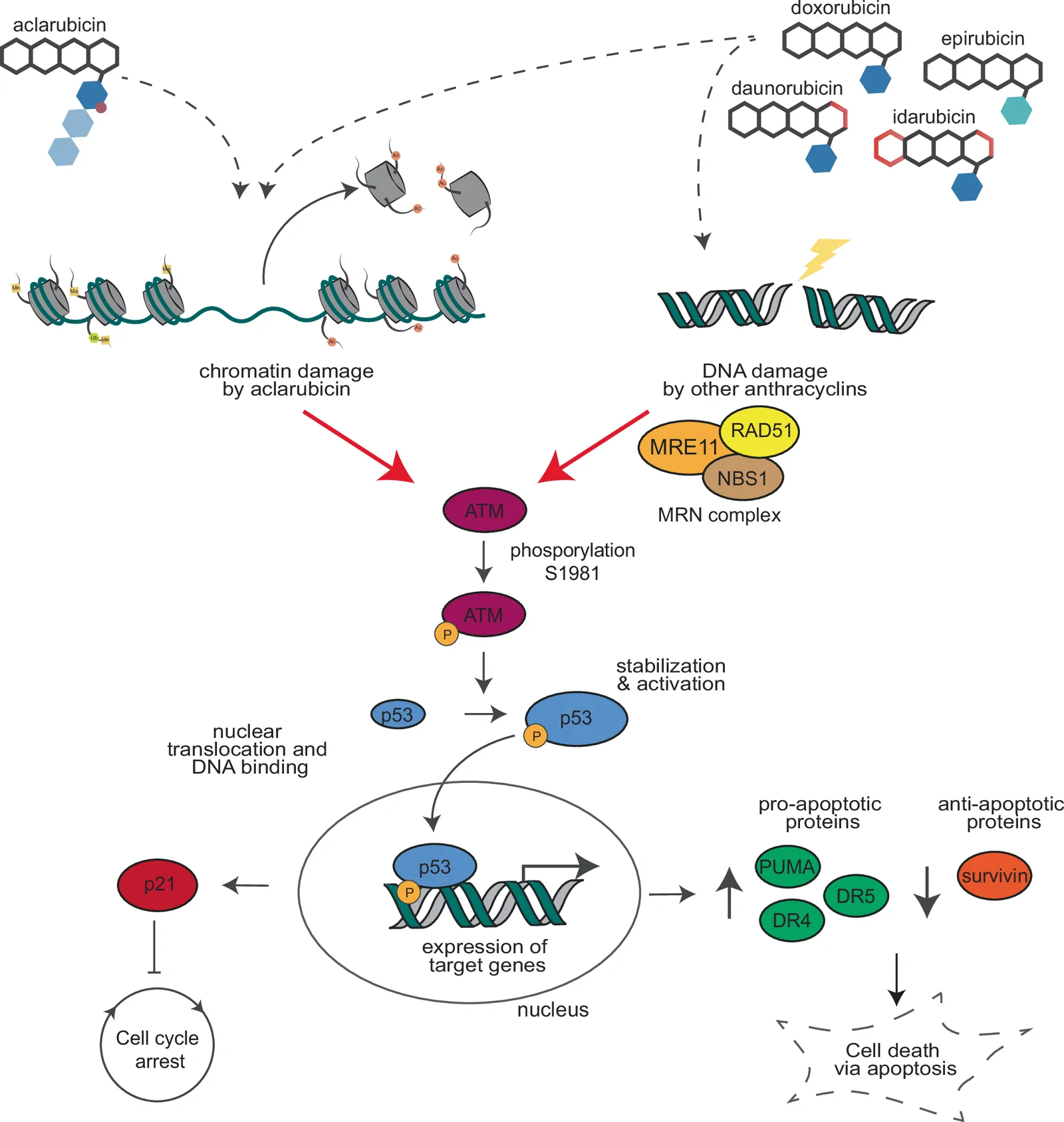
Model of p53-mediated apoptosis upon anthracycline treatment. Topoisomerase II poisons like doxo-, dauno-, epi- and idarubicin introduce DNA damage via the formation of topoisomerase-DNA complexes. These drug-mediated DNA breaks subsequently lead to p53 activation. The histone-evicting anthracycline aclarubicin can also induce p53 relocalization and activation via ATM activation/phosphorylation followed by induction of downstream signaling. Clinical anthracycline drugs (with both DNA- and chromatin damage) thus activate p53-dependent apoptosis and cell cycle arrest by two different mechanisms.

To further investigate the potential mediators of aclarubicin-induced cell death upstream of p53, we examined ATM and ATR activation over time in p53-proficient and -deficient 93.05 cells following aclarubicin exposure (Fig. 5A–C). ATM phosphorylation increased rapidly after aclarubicin exposure in both p53-proficient and -deficient cells (Fig. 5A, B). In contrast, ATR activation occurred at a later time point and was higher in the p53-deficient cells (Fig. 5A, C). To investigate whether aclarubicin treatment affects the association of ATM and/or ATR to DNA, as also observed for p53 (Fig. 3D, E), we performed a chromatin association assay (Fig. S7). Neither ATM nor ATR associated strongly with DNA after aclarubicin or doxorubicin exposure, suggesting that histone eviction does not promote DNA binding of ATM or ATR. To determine whether ATM activity controls activation of p53, we used ATM inhibitor KU0055933 and measured whether inhibition of ATM would reduce p53 activation after aclarubicin exposure (Fig. 5D). Indeed, pre-treatment with KU0055933 reduces both ATM as well as p53 activation and, to a lesser extent, p53 stabilization (Fig. 5D-G). To evaluate the role of ATR, we repeated the experiment in the presence of the ATR inhibitor VE-821 and subsequently evaluated p53 activation after aclarubicin exposure (Fig. 5H). While ATR inhibition appeared to mildly reduce p53 activation, the effect was smaller when compared to that of ATM inhibition and did not impact p53 stabilization (Fig. 5K, L). Finally, we performed a survival assay to determine whether ATM or ATR mediates aclarubicin-induced cell death in p53-proficient and -deficient cells (Fig. 5M). We observed that ATM, but not ATR, inhibition significantly increases survival of p53-proficient, but not p53-deficient cells. Together, these observations suggest that aclarubicin-induced chromatin damage activates ATM, which in turn induces p53 activation leading to cell death (and cell cycle arrest) (Fig. 6).

## Discussion

Aclarubicin can be considered a distinct member of the anthracycline family due to its inability to poison Topoisomerase II and inflict DNA double-strand breaks. We and others have recently shown that its ability to evict histones is critical to its anti-cancer activity [6, 8, 9]. How this activity translates into cell death is unclear. Using CRISPR-screening, we identified and established a key role for p53 in this process, where cellular sensitivity can be predicted by p53 status. Furthermore, we demonstrated here that aclarubicin activates a p53-dependent transcriptional program inducing cell cycle arrest and apoptosis similar to DNA-damaging compounds. Thus, even in the absence of DNA double-stranded break formation, chromatin damage induced by aclarubicin treatment can activate p53 and induce cell death. As a consequence, regular topoisomerase II-poisoning anthracyclines appear to engage two distinct mechanisms leading to p53 activation and ultimately apoptosis and cell cycle arrest; one initiated by the DNA damage response (as is the case for DNA damage-inducing variants such as doxorubicin and daunorubicin) and the other initiated by the chromatin damage response as exemplified by aclarubicin. We demonstrated that histone eviction leads to both ATM and ATR activation over time, yet only ATM activation appears to be crucial for p53 activation. The mechanism by which histone eviction activates ATM remains to be elucidated, but it is independent of the MRN complex. In the absence of p53, apoptosis is still observed, but at greatly reduced efficiency. Regarding cell cycle arrest, we still observe cells accumulating in G2/M phase, but previous findings suggest these cells are likely stuck in mitosis rather than undergoing cell cycle arrest [20]. A second CRISPR screen failed to yield candidate hits that could explain p53-independent cell death and cell cycle arrest by aclarubicin. Together, these findings imply that multiple chromatin damage sensing mechanisms might be in play.

Our findings that chromatin damage activates p53 and induces apoptosis are in line with reports about other chromatin-damaging agents such as the curaxins [21, 22]. Curaxins can trap the FACT complex on DNA, leading to activation of casein kinase 2(CK2) and thereby phosphorylation of p53 at Ser392 but not Ser15. Aclarubicin treatment also induces FACT binding to DNA [22], suggesting it could also activate CK2. However, we observed increased phosphorylation of p53 at Ser15 after aclarubicin exposure, suggesting that chromatin damage induced by aclarubicin has additional mechanisms that are not observed after curaxin exposure. The existence of multiple chromatin damage sensing mechanisms would also explain why we failed to identify any proteins of the FACT/CK2 pathway in the CRISPR screen. Upon p53 activation, several downstream signaling cascades are activated, leading to apoptosis. Whilst we observe p53-dependent apoptosis and increased cell death upon addition of apoptosis-activators, other forms of cell death are also induced by aclarubicin. Blocking apoptosis by the apoptosis inhibitor Z-VAD-FMK did not render cells significantly more resistant to aclarubicin (Fig. S6). Thus, while p53-dependent apoptosis might be the dominant mechanism of cell death, in its absence cells will employ one or multiple pathways also resulting in drug-induced cell death.

It has been reported that in contrast to doxorubicin, aclarubicin can sensitize cells to tumor necrosis factor-related apoptosis-inducing ligand (TRAIL) induced apoptosis [23]. While in our screen DR4 was one of the top hits and we observed upregulation of both DR4 and DR5 after exposure to aclarubicin, in our setting the induction of both genes was dependent on p53, suggesting that there might be factors other than p53 that enable upregulation of DR5 expression in certain circumstances. Aclarubicin treatment has also other effects such as inhibition of transcription and inhibition of protein degradation [24–27]. These experiments were often done at higher concentrations, suggesting they may be more relevant at high dosages and/or in a p53-deficient setting.

Aclarubicin is mainly used for the treatment of acute myeloid leukemia (AML), as part of the CAG regimen (cytarabine, aclarubicin and G-CSF), both as first-line treatment and for relapsed cases [14, 28, 29]. Our data suggest that p53-mutations would be a good stratification method for aclarubicin treatment to identify which patients would benefit and simultaneously prevent patients from receiving ineffective treatment. While p53 is mutated in only about 5–10% of the de novo AML cases, it is more frequently observed in therapy-resistant AML (25–33%) [30–33]. In both cases, loss of p53 is strongly correlated with poor outcome and prognosis. Unfortunately, these patients will probably benefit less from an aclarubicin-based treatment, and other treatments would have to be developed, surpassing the prerequisite for p53. Alternatively, aclarubicin could still be effective in inducing cell death of p53-deficient cells, but requiring higher concentrations. This could be a basis for ‘high-dose chemotherapy’. As aclarubicin lacks many treatment-limiting side effects [8, 14], using it at a higher dose can be considered. The stratification of the p53 status of AML and other tumors may be a rationale for normal or high-dose chemotherapies in the treatment of cancer patients. Recently, the BCL-2 inhibitor venetoclax (ABT-199) was approved as first-line treatment for AML, which is particularly useful for patients who are unable to tolerate classical intense chemotherapy, including elderly patients (>75 years old). Our data show that aclarubicin synergizes with ABT-199 to increase cytotoxicity, indicating that p53 wild-type AML patients could potentially benefit from the combination therapy, with limited toxicity due to low adverse effects of both drugs.

In conclusion, we identified p53 as a major determinant in cellular sensitivity towards the chromatin-damaging anthracycline drug aclarubicin. While aclarubicin does not induce DNA double-strand breaks, unlike other clinically applied anthracycline family members, the chromatin damage induced by these drugs induces ATM activation followed by p53 relocalization and activation, resulting in p53-dependent apoptosis and cell cycle arrest.

## Materials and methods

### Reagents

Aclarubicin (sc-200160) was purchased from Santa Cruz Biotechnology (USA) and dissolved in dimethylsulfoxide at 5 mg/ml concentration, aliquoted, and stored at −20 °C for further use. Doxorubicin was obtained from Pharmachemie (the Netherlands). HDM201 and RG7112 were obtained from (Selleckchem), Venetoclax/ABT-199 (Tocris), KU-0055933, MIK665 and VE-821 (MedChemExpress).

### Cell culture

MelJuSo cells were cultured in IMDM supplemented with 8% fetal calf serum, and cell line authentication was performed by Eurofins Genomics (19-ZE-000487). HEK293T cells (obtained from ATCC (CRL-3216)) and the cutaneous melanoma cell line 93.05, a gift from Sjoerd van den Burg and Els Verdegaal (LUMC, Leiden, The Netherlands), were cultured in DMEM supplemented with 8% fetal calf serum. MEL202 cells were grown in a mixture of RPMI/DMEM-F12 + 10% FCS. The MEL202 cell line was provided by Dr. Bruce Ksander, Dept. Ophthalmology, Harvard University, Boston, USA. The generation of monoclonal p53-KO derivatives of 93.05 and MEL202 cells has been described [34]. HCT116-Ctrl and p53KO cells were a gift from Bert Vogelstein and grown in DMEM/10% FCS [20]. The RPE1-Ctrl and p53KO cells were a gift from Job de Lange and grown in DMEM + 10% FCS [35]. p53-wildtype (A549, FM3, MelJuSo, U2OS, U87) and p53-mutant (BT474, BXPC3, DU145, K562, PC3, SKBR3 and U118) cells were cultured and tested as described before [10]. All cell lines were maintained in a humidified atmosphere with 5% CO_2_ at 37°C and regularly tested for the absence of mycoplasma.

### CRISPR knockout screen

For knockout screening, the human CRISPR Brunello genome-wide knockout library was a gift from David Root and John Doench (Addgene #73178). Two batches of 1×10^8^ MelJuSo or MelJuSo sg*TP53*-1 melanoma cells were infected at an MOI of 0.3. Transduced cells were selected using puromycin (1 µg/ml) for five days. After the selection procedure, cells were treated continuously with aclarubicin at a concentration of 100 nM MelJuSo or 250 nm (MelJuSo sg*TP53*-1) for 9 days. After this, cells were grown out for an additional 3 days, and genomic DNA was isolated from the aclarubicin-treated cells, as well as from the control population that was grown in parallel. sgRNAs were amplified using the established protocol [36]. gRNAs were sequenced using the Illumina NovaSeq6000, and inserts were mapped to the reference. Statistical analysis was done using RSA analysis; enrichment >2.5 was considered a candidate hit [37].

### Transductions

For the generation of viral particles, HEK293T cells were transfected using polyethyleneimine (Polyscience Inc.) with packaging plasmids pRSVrev, pHCMV-G VSV-G and pMDLg/pRRE in combination with the lentiviral construct. Virus was harvested, filtered, and target cells were transduced in the presence of 8 µg/ml polybrene (Millipore).

### Cloning and subclone generation

For p53 validation of the knockout screen, two individual guides were cloned into the LentiCRISPR v2 vector (a gift from Feng Zhang, Addgene plasmid #52961) and the indicated cells were transduced and selected using puromycin. Guide sequences were as follows: p53-1: 5’-CCATTGTTCAATATCGTCCG-3’, p53-2: 5’-GGTGCCCTATGAGCCGCCTG-3’. For genomic validation, genomic DNA was isolated, and a PCR fragment of ±300 bp was cloned into an empty GFP-C1 vector (Clonetech) using NEBBuilder HiFi fidelity assembly mix using the primers listed in Supplementary Table 3: Sequences were then verified using primers listed in Supplementary Table 3. The sgRNAs for MRE11A (5’-GTTTGCTGCGTATTAAAGGG-3’) and NBN (5’-CCAGGACCAAGCCTTTCACA-3’) were cloned into the PX459 vector. MelJuSo cells were transfected with the sgRNA vectors using Lipofectamine 2000 and selected with puromycin, followed by subcloning. Clones were validated for complete knockout using Western Blot.

### Long-term proliferation assays

Cells were seeded into 12-well plates (5000 cells/well). The next day, aclarubicin was added at the indicated concentrations and incubated for 2 hours. Subsequently, cells were washed extensively and left to grow for 7 to 9 days. For fixing and staining, cells were washed with PBS and incubated for 20 min at RT with Crystal violet staining solution (0.05% w/v Crystal Violet, 1% formaldehyde, 1% methanol in 1X PBS). Quantification of colonies was done by extraction of the crystal violet. To do so, colonies were incubated for 20 min at room temperature with 15% acetic acid (500 µL). Readout was done by measuring the absorbance using a CLARIOstar plate reader (BMG Labtech).

### Short-term growth assays

Cells were seeded into 96-well plates and exposed the next day to indicated drugs at different concentrations. When indicated, drugs were removed two hours later and cells were cultured for an additional 72 hours. Cell viability was measured using the CellTiter-Blue viability assay (Promega). Relative survival was normalized to the untreated control and corrected for background signal.

### Apoptosis assay

Cells were seeded in triplicate in white-walled 96-well plates with clear bottoms and in clear 96-well plates. The next day, the cells were exposed to the indicated compound(s). Three days later, caspase 3/7 activity was assessed with the use of the Caspase-Glo 3/7 Assay (Promega), and cell viability was assessed with the CellTiter-Blue Cell Viability Assay. Relative caspase activity was normalized to cell number.

### Chromatin association assay

Cells were seeded into 6 cm dishes (1×10^6 cells/well). The next day, indicated drugs were added for 30 min at 10 µM final concentration. Cells were lysed in lysis buffer (25 mM HEPES pH 7.6, 5 mM MgCl_2_, 25 mM KCl, 0.05 mM EDTA, 10% glycerol, 0.1% NP-40) for 30 minutes at 4 °C, and nuclei were spun down and resuspended in 75 µL buffer (20 mM Tris-HCl pH 7.6, 3 mM EDTA). Of this, 25 µL samples were adjusted to the indicated NaCl concentrations to a total volume of 50 µL and incubated for 20 min. On ice. Subsequently, chromatin was spun down and resuspended in SDS-sample buffer (2% SDS, 10% glycerol, 5% β-mercaptoethanol, 60 mM Tris-HCl pH 6.8, and 0.01% bromophenol blue), after which samples were analyzed by SDS-PAGE and Western blotting.

### Cell cycle analysis

Cells were seeded in a 6-well plate and exposed to 200 nM aclarubicin once they reached 60% confluency for 2 hours, followed by washing and left to grow for another 22 hours. Cells were then collected and stained with a hypotonic PI staining buffer with 40 µg/mL RNAse A [38] for 10 min at RT in the dark. Samples were transferred to ice and incubated for a further 30 min and run on a LSR II flow cytometer (BD Biosciences). The PI staining was analyzed using the cell cycle tool in FlowJo version 10 using the unconstrained Watson model.

### Western blot

Cells were treated with the indicated concentration of aclarubicin for 2 hours, followed by washing, or other drugs continuously at the indicated concentrations. Cells were collected and lysed directly in SDS-sample buffer (2% SDS, 10% glycerol, 5% β-mercaptoethanol, 60 mM Tris-HCl pH 6.8, and 0.01% bromophenol blue). Lysates were resolved by SDS-PAGE followed by Western blotting. Primary antibodies used for blotting: p53 (DO-1, sc-126 Santa Cruz), γH2AX (1:1000, 05-036, Millipore), β-actin (A5441, Sigma), pS15p-53 (#9284, Cell Signaling Technology), pS1981-ATM (Clone D25E5 #13050 Cell Signaling Technology), ATM (Clone Y170; ab32420 Abcam), ATR (sc-515173, Santa Cruz), pT1989 ATR (Clone D5K8W, #30632 Cell Signaling Technology), pS842-KAP1 (A300-767A; Bethyl Laboratories), KAP1 (A300-274A; Bethyl Laboratories), Mdm2 (Clone 3G9; 04-1530, Merck Millipore), PUMA (Clone G3; sc-374223, Santa Cruz Biotechnology), p21 (clone CP74; 05-655-MI Merck Millipore), PARP (#9542, Cell Signaling Technology), Vinculin (clone hVIN-1; V9131, Sigma-Aldrich), MRE11 (clone 1D12; GTX70212 Genetex), NBS1 (clone 1C3; MA1-23263 Thermo Scientific). Images were quantified with ImageJ. Uncropped WB can be found in the supplementary material.

### Microscopy

For endogenous p53 staining cells were seeded on coverslips, and the next day exposed for the indicated time with 200 nM aclarubicin. Upon treatment, cells were fixed in 4% formaldehyde, permeabilized with 0.1% Triton, blocked in 0.5% BSA and stained with mouse monoclonal anti-p53 (DO1, sc-126, Santa Cruz), goat-anti-mouse-Alexa-Fluor-488 (Thermo Fisher Scientific), Alexa-Fluor-647-phalloidin (A22287, Thermo Fisher Scientific) and DAPI. Cells were analyzed by a Leica SP8 confocal microscope system with 63x lens. Cells stably expressing PAGFP-H2A were used for histone eviction experiments. Photoactivation and time-lapse confocal imaging were performed as described [6] and loss of fluorescence from the photoactivated region after different treatments was quantified. Quantification was done using ImageJ software.

### RT-PCR

RNA was isolated using the SV total RNA isolation kit (Promega), after which cDNA was synthesized using the reverse transcriptase reaction mixture as indicated by Promega. qPCR was performed using SYBR green mix (Roche Diagnostics, Indianapolis, IN, USA) in a C1000 touch Thermal Cycler (Bio-Rad Laboratories, Hercules, CA, USA). In independent experiments, the expression of target genes was determined and normalized to at least two housekeeping genes, *CAPNS1* and *SRPR*. Primers for detection are listed in Supplementary Table 3.

### Data and statistical analysis

For in vitro experiments, each sample was assayed in biological triplicate, unless stated otherwise. No statistical methods were used to predetermine sample size. All error bars denote mean + SD. Statistical analyses were performed using Prism 10.2 software (Graphpad Inc). Western blot and confocal data were quantified using ImageJ software. Significance is represented on the graphs as follows: ns, not significant, *p < 0.05, **p < 0.01, ***p < 0.001, ****p < 0.0001.

## Supplementary information

Supplementary Table 1

Supplementary Table 2

Supplementary Table 3

Van der Zanden et al. Supplementary Information

Uncropped western blots

## Data Availability

Data generated in this study is available upon request from the corresponding authors.
