# Supplementary material for: Genome-wide CRISPR-screening identifies p53 as regulator of cancer cell sensitivity to the histone evicting anthracycline aclarubicin
Source: Cell Death Dis. 2026 Aug 5;17(1):933. doi: 10.1038/s41419-026-09138-6 (PMC13728235; doi:10.1038/s41419-026-09138-6)
Supplement: Supplementary file 1 [file 41419_2026_9138_MOESM4_ESM.pdf]

Genome-wide CRISPR-screening identifies p53 as regulator of cancer cell sensitivity to the histone evicting anthracycline aclarubicin

Van der Zanden et al.

Supplementary Information

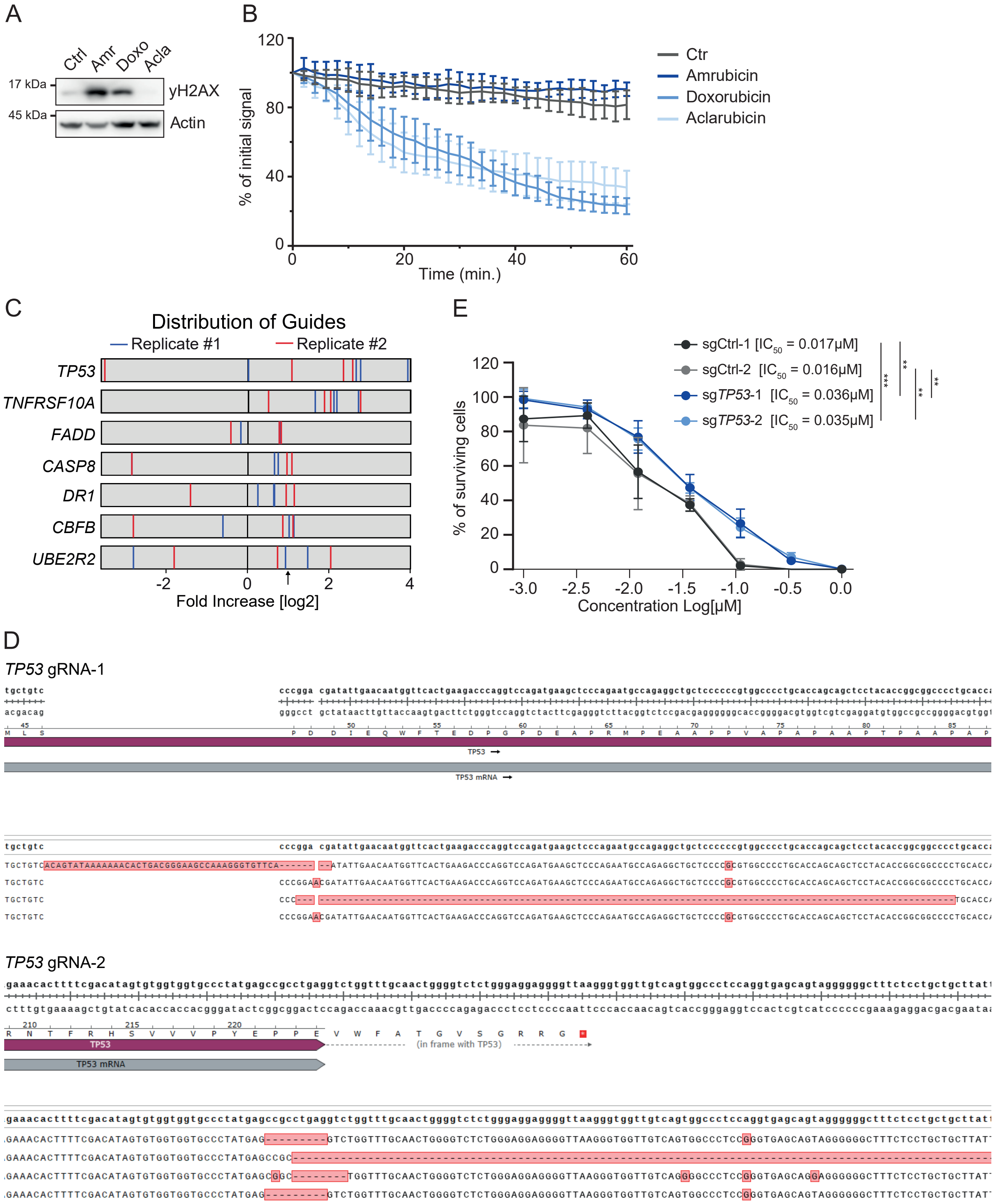

Fig. S1 p53 as a regulator of sensitivity to aclarubicin. A Western blot showing DNA double strand breaks induced by three different anthracycline drugs in MelJuSo cells treated for 2hr with 10 μM of the drugs. Actin was used as a loading control. Corresponding to quantification in Fig. 1A, bottom panel. **B** Fluorescent signal of photo-activated GFP-H2A histones was followed in living cells over time upon treatment with the different drugs. Quantification of loss of initial signal upon histone eviction is plotted. Corresponding to quantification of EC_{50} in Fig. 1A, bottom panel. **C** gRNA enrichment for hits with at least two guides per sort enriched >2.5-fold. Arrow depicts 2.5-fold threshold and red and blue stripes represent enrichment of individual gRNAs compared to the non-sorted population. **D** Snapshot of Snapgene, showing genomic validation of pooled MelJuSo p53-knock out cell lines. **E** Indicated MelJuSo cells were treated for 72h with various concentrations of aclarubicin followed by assessing the cell viability. *n* = 3 independent experiments. IC_{50} for each cell line is indicated. Two-tailed *t*-test, ** *p*< 0.01, *** *p*< 0.001.

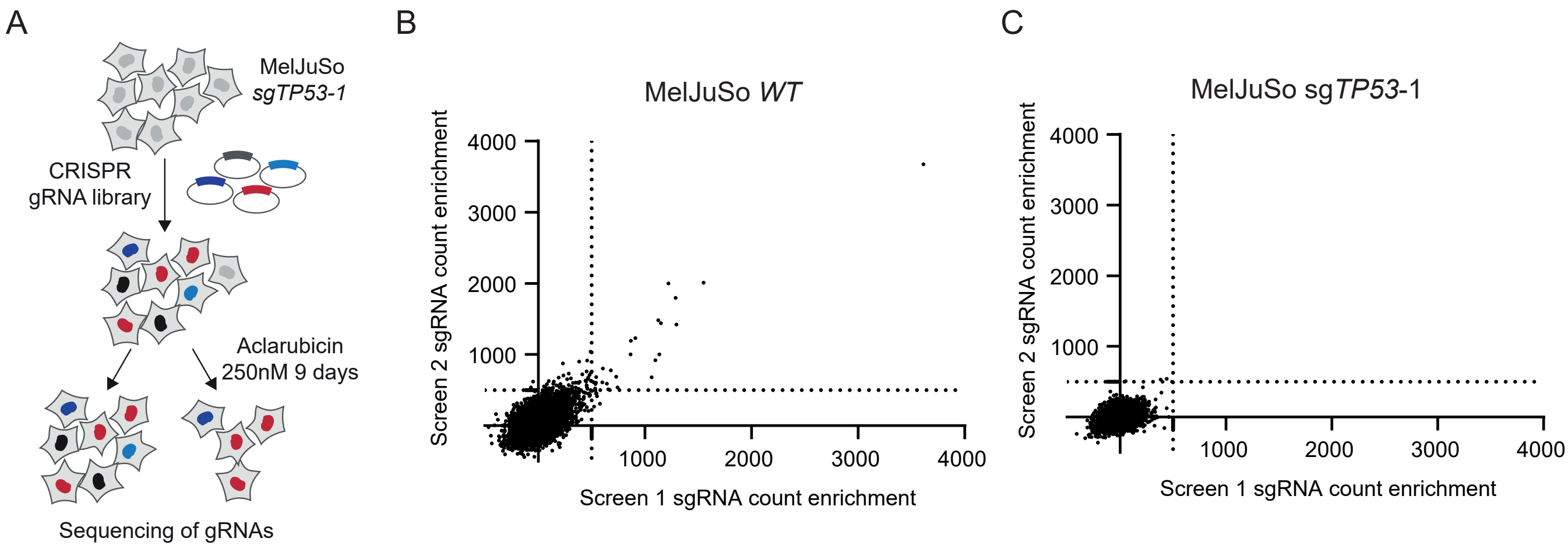

Fig. S2 genome-wide CRISPR screening in p53-deficient background yields no additional hits. **A** Schematic representation of the screening outline. MelJuSo sg*TP53*-1 melanoma cells were transduced with the Brunello genome-wide CRISPR knockout library and cells were either or not treated with aclarubicin (250 nM) for 9 days followed by gRNA sequencing. **B-C,** Graphs depicting the count enrichment of individual sgRNA's in the two replicate screens in WT MelJuSo (**B**) and MelJuSo sg*TP53*-1 (**C**) cells illustrating the lack of enriched sgRNA's in the p53-deficient cells.

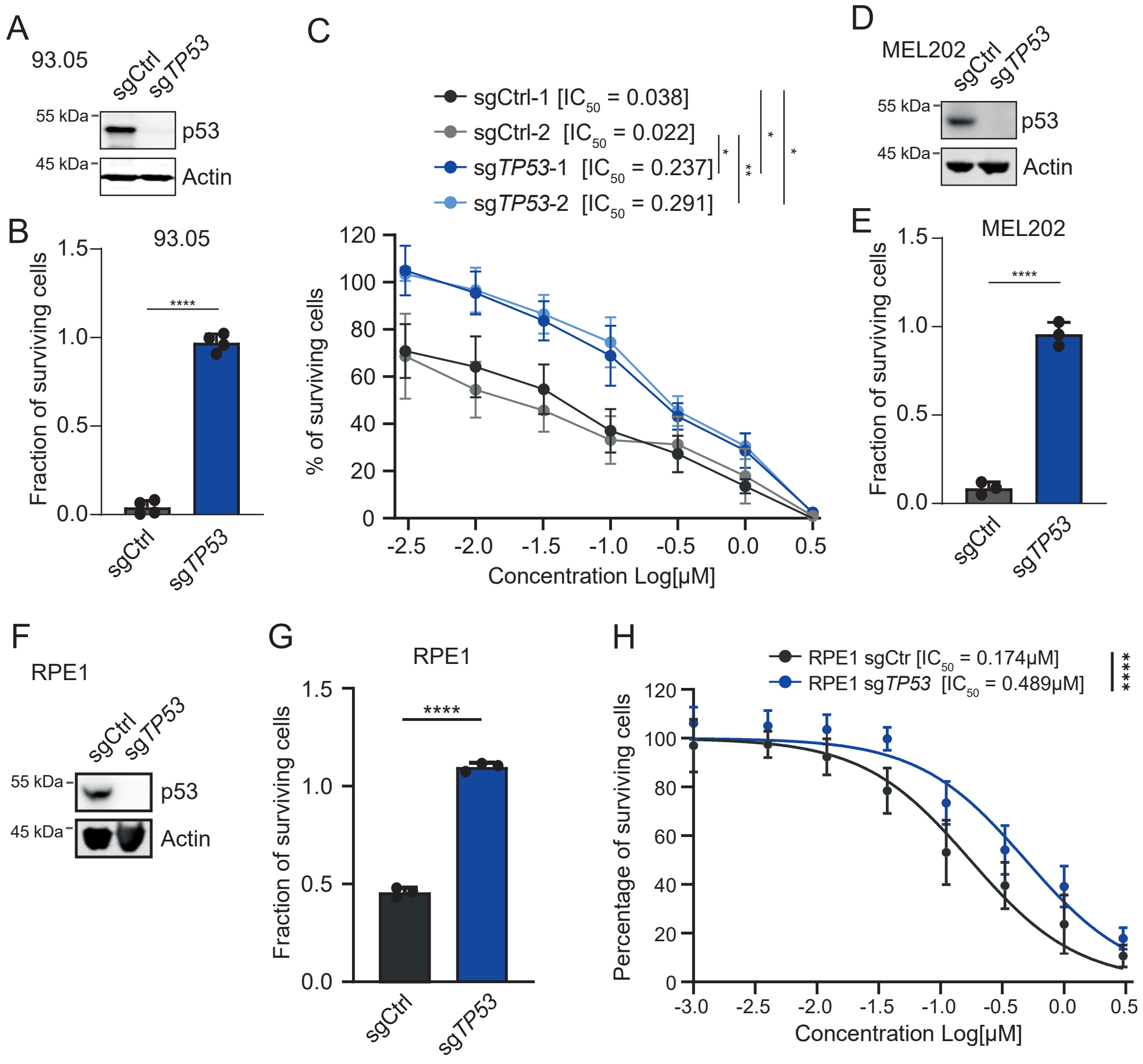

Fig. S3 p53 as a major determinant in sensitivity to Aclarubicin. A 93.05 cells were analyzed for p53-expression using Western blot. **B** 93.05 cells as in **A** were treated with HMD201 for 3 days and viability was assessed and normalized to untreated cells. *n* = 4 independent experiments. Unpaired t-test, **** *p*< 0.0001. **C** Indicated 93.05 cells were treated for 2h with various concentrations of aclarubicin after which the drug was removed by washing and cells were left to grow for another 3 days before assessing cell viability. *n* = 3 independent experiments. IC_{50} for each cell line is indicated. Two-tailed *t*-test, * *p*< 0.05, ** *p*< 0.01. **D** MEL202 cells were analyzed for p53-expression using western blot. **E** MEL202 cells as in **A** were treated with HMD201 for 3 days and viability was assessed and normalized to untreated cells. *n* = 3 independent experiments. Unpaired *t*-test, **** *p*< 0.0001. **F.** RPE1 (hTERT-immortalized retinal pigment epithelial) cells were transduced with the indicated CRISPR constructs and analyzed for p53-expression using western blot. **G** RPE1 cells as in **F** were treated with HMD201 for 3 days and viability was assessed and normalized to untreated cells. *n* = 3 independent experiments. Unpaired t-test, **** p< 0.0001. **H.** RPE1 sgCtr and sg*TP53* cells were treated for 2h with various concentrations of aclarubicin after which the drug was removed by washing and cells were left to grow for another 3 days before assessing cell viability. Unpaired *t*-test, **** p< 0.0001.

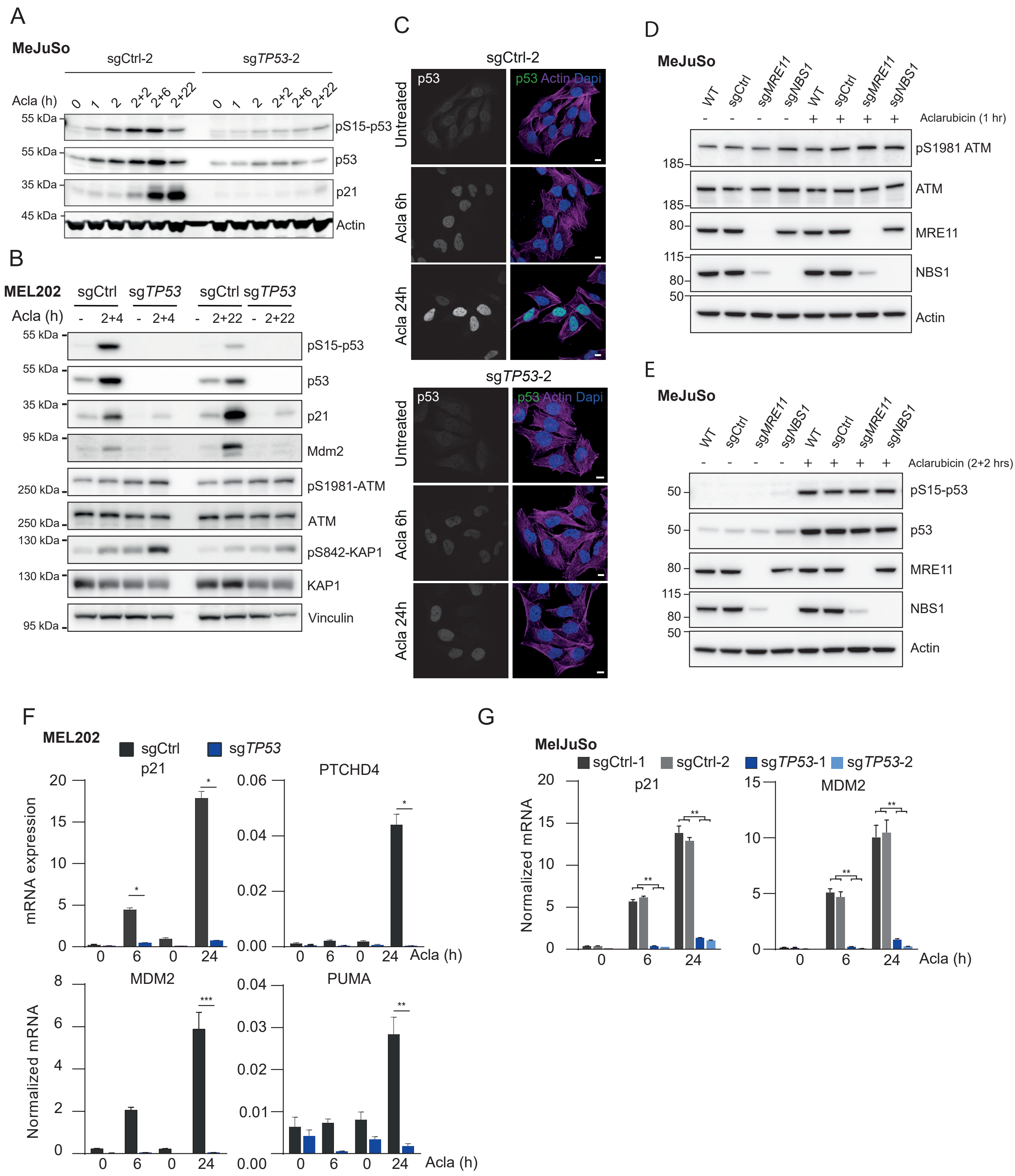

A
MeJuSo
sgCtrl-2
sgTP53-2
Acla (h)
0 1 2 2+2 2+6 2+22
pS15-p53
p53
p21
Actin
B
MEL202
sgCtrl sgTP53 sgCtrl sgTP53
Acla (h)
pS15-p53
p53
p21
Mdm2
pS1981-ATM
ATM
pS842-KAP1
KAP1
Vinculin
C
sgCtrl-2
sgTP53-2
p53
p53 Actin Dapi
Untreated
Acla 6h
Acla 24h
D
MeJuSo
WT sgCtrl sgMRE11 sgNBS1
Aclarubicin (1 hr)
pS1981 ATM
ATM
MRE11
NBS1
Actin
E
MeJuSo
Aclarubicin (2+2 hrs)
pS15-p53
p53
MRE11
NBS1
Actin
F
MEL202
sgCtrl sgTP53
p21
PTCHD4
MDM2
PUMA
mRNA expression
Normalized mRNA
Acla (h)
G
MelJuSo
sgCtrl-1 sgCtrl-2 sgTP53-1 sgTP53-2
p21
MDM2
Normalized mRNA
Acla (h)

Fig. S4 Aclarubicin induces nuclear accumulation and activation of p53 independent of the MRN complex. A MelJuSo cells were exposed to 200 nM aclarubicin for the indicated time points and washed away after maximum 2h, after which cells were left to recover for the indicated time points before lysis and analysis of the indicated proteins by Western blot. **B** MEL202 melanoma cells were exposed to aclarubicin for 2h, washed and lysed 4h or 22h later, before analysis of the indicated proteins by Western blot. **C** Representative confocal images of fixed MelJuSo cells stained for endogenous p53. Cells were treated with 200 nM aclarubicin for 2h, washed and fixed at the indicated time points post treatment. DAPI and actin are stained as nuclear and cytosolic marker, respectively. Scale bar; 10 μm. **D,** MelJuSo cells were exposed to 200 nM aclarubicin for 1 hour before lysis and analysis of the indicated proteins by Western blot. **E** MelJuSo cells were exposed to 200 nM aclarubicin for 2 hours after which cells were left to recover for 2 hour before lysis and analysis of the indicated proteins by Western blot. **F** MEL202 cells were exposed for 2h to 200 nM aclarubicin and RNA was isolated 4h (6h) or 22h (24h) post treatment. Expression of indicated mRNA was detected using qRT-PCR and normalized to housekeeping gene expression. n = 2 independent experiments, data represent technical triplicates. Statistical significance was measured using a Student's t-test, * p<0.05, ** p< 0.01. **G** Indicated MelJuSo cells were exposed for 2h to 200 nM aclarubicin and RNA was isolated 4h (6h) or 22h (24h) post treatment. Expression of indicated mRNA was detected using qRT-PCR and normalized to housekeeping gene expression. n = 2 independent experiments, data represent technical quadruplicates. Statistical significance was determined by a Student's t-test, ** p< 0.01.

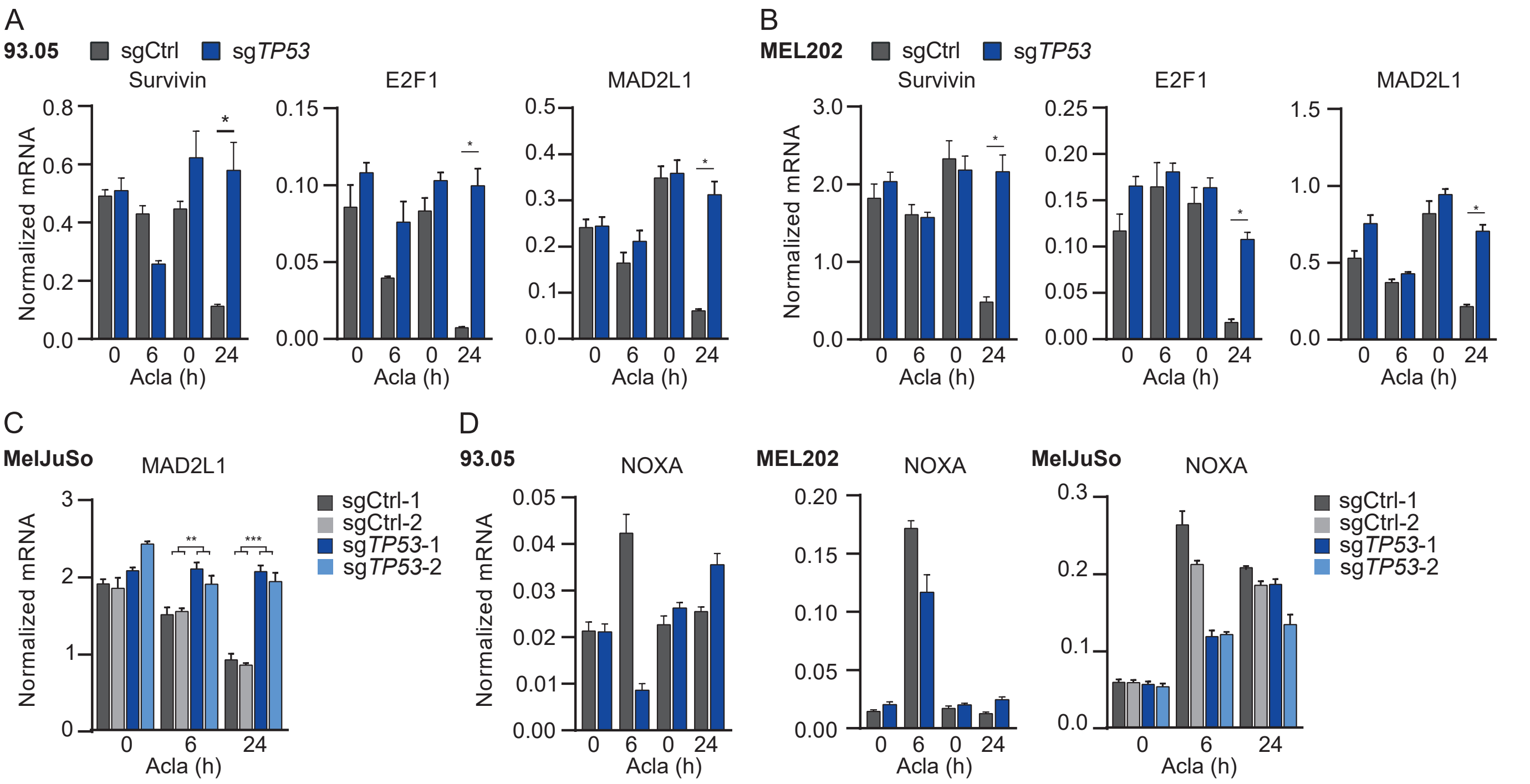

Fig. S5 Aclarubicin induces p53-dependent decrease of surviving, E2F1 and MAD2L1 expression. Indicated cells were treated with 200 nM aclarubicin for 2h and RNA was isolated 4h (6h) or 22h (24h) post treatment. Expression of indicated mRNA was detected by qRT-PCR and normalized to housekeeping gene expression. *n* = 2 independent experiments, data represent quadruplicates triplicates. Statistical significance was determined by a Student's *t*-test, * p<0.05.

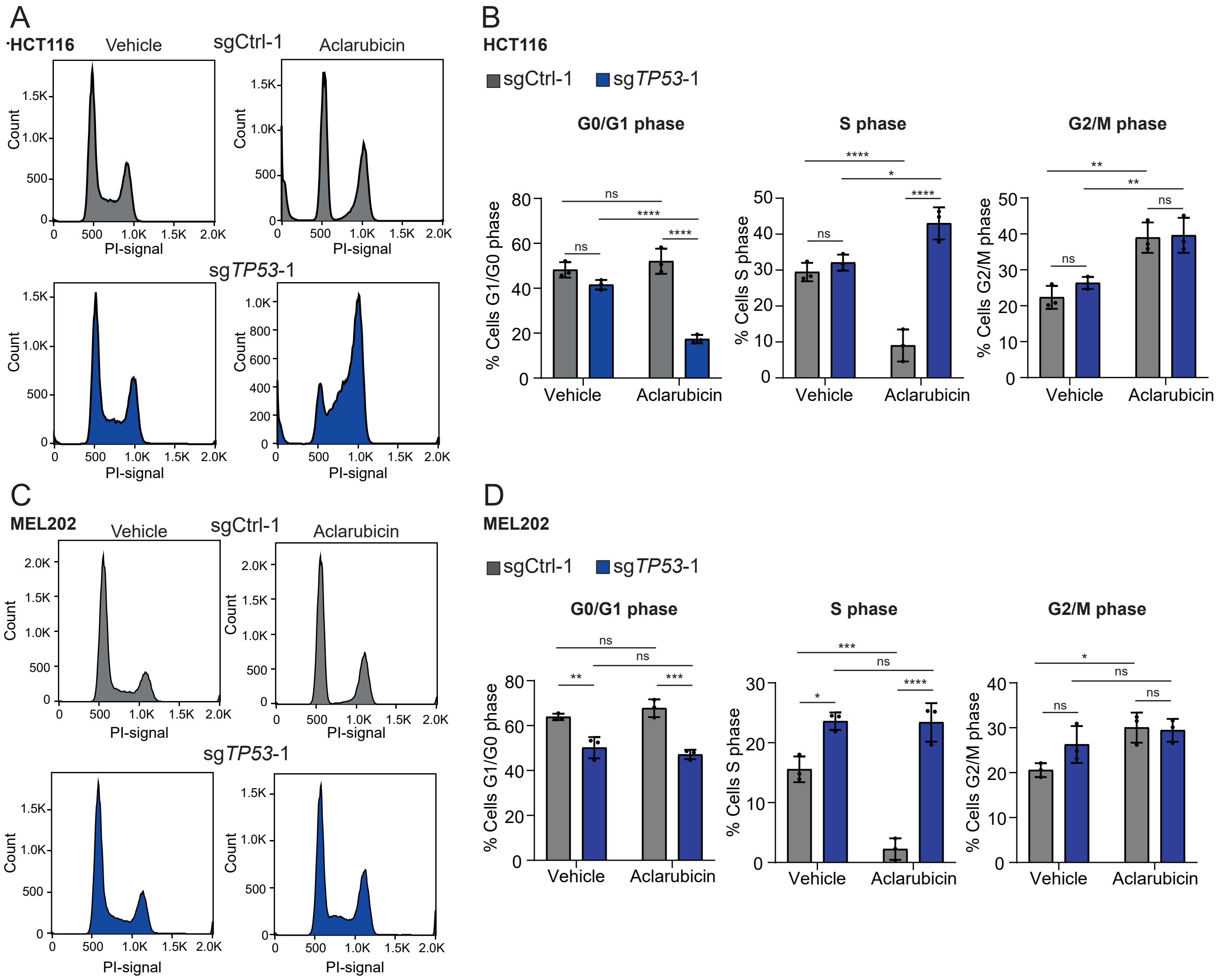

Fig. S6 Aclarubicin induces p53 dependent cell cycle arrest. A-D Representative histograms (**A, C**) and quantifications (**B, D**) of propidium iodide staining of HCT116 (**A**, **B**) and MEL202 (**C**, **D**) sgCtrl-1 and sg*TP53*-1 cells exposed to 200 nM aclarubicin for 2 hours and collected 22 hours later. Data represent three independent experiments. Two-way ANOVA with multiple comparisons, * $p< 0.05$, ** $p< 0.01$ *** $p< 0.001$, **** $p< 0.0001$.

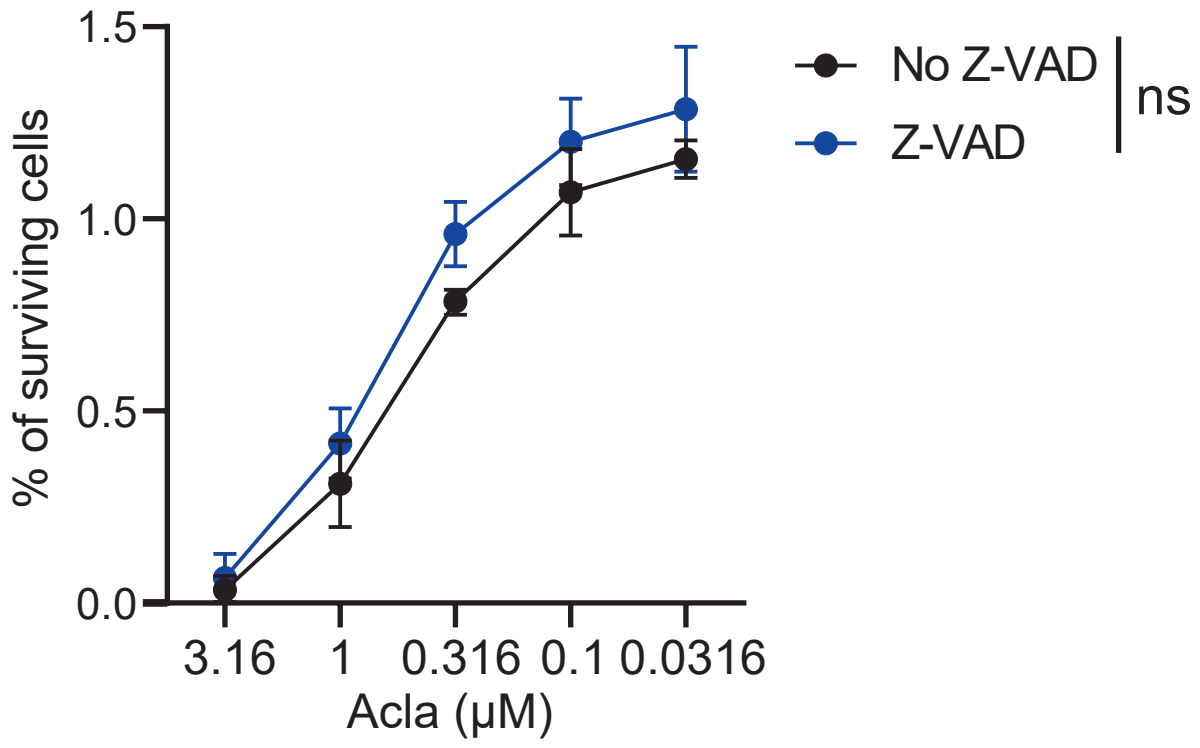

Fig. S7 Z-VAD does not significantly affect resistance to aclarubicin. MelJuSo cells were treated for 2h with different concentrations of aclarubicin, drug was removed by washing and cells were left to grow out in the presence or absence of Z-VAD-FMK. Three days later, cell viability was measured and normalized to the non-aclarubicin treated controls. Data represent two independent experiments. Statistical significance was determined by a two-way ANOVA with multiple comparisons, ns is not significant.

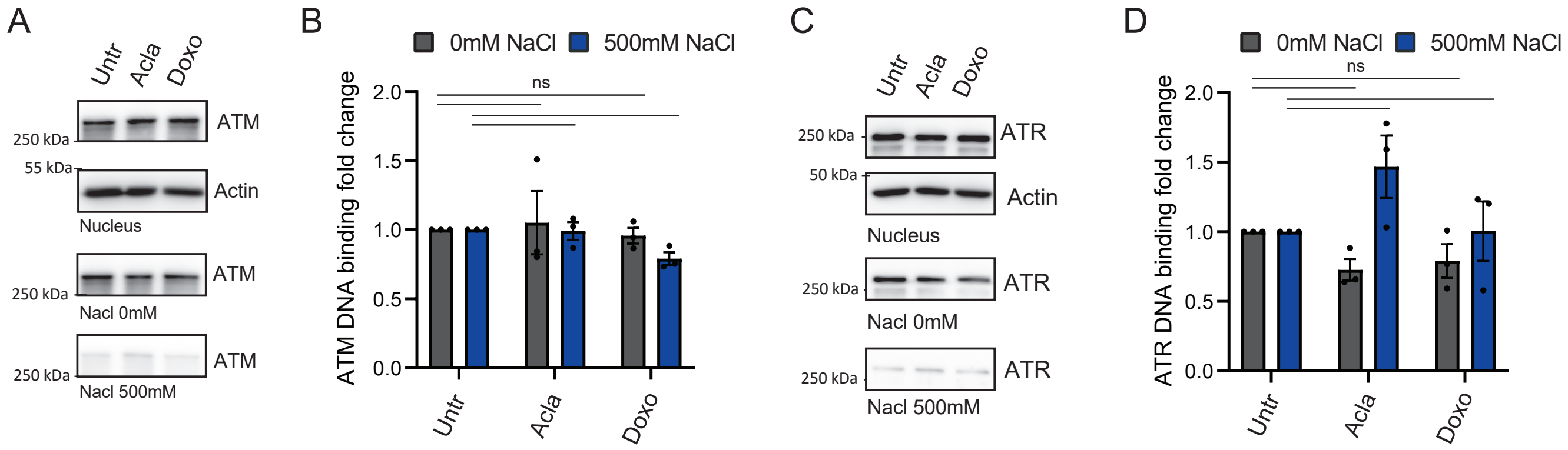

Fig. S8 Aclarubicin does not alter DNA binding of ATM or ATR. A Chromatin binding assay for endogenous ATM in MelJuSo cells. Cells were treated with 10 µM doxo- or aclarubicin for 45 minutes followed by fractionation. ATM localization and DNA binding was analyzed by Western Blot. **B** Quantification of nuclear ATM localization. Ordinary two-way ANOVA with multiple comparisons, ns is not significant. **C** Chromatin binding assay for endogenous ATR in MelJuSo cells. Cells were treated with 10 µM doxo- or aclarubicin for 45 minutes followed by fractionation. ATR localization and DNA binding was analyzed by Western Blot. **D** Quantification of nuclear ATR localization. Ordinary two-way ANOVA with multiple comparisons, ns is not significant.

Supplementary Table 1 (external file)

sgRNA Counts and fold changes of CRISPR screens in WT (first tab) and p53-deficient (second tab) MelJuSo cells in the presence and absence of Aclarubicin.

Supplementary Table 2 (external file)

TP53 mutational status and aclarubicin sensitivity (IC50) of cell lines used in Figure 2G

Supplementary Table 3 (external file)

List of primers used for molecular cloning and qPCR analysis
