# Supplementary material for: Genome-wide CRISPR-screening identifies p53 as regulator of cancer cell sensitivity to the histone evicting anthracycline aclarubicin
Source: Cell Death Dis. 2026 Aug 5;17(1):933. doi: 10.1038/s41419-026-09138-6 (PMC13728235; doi:10.1038/s41419-026-09138-6)
Supplement: Supplementary file 2 [file 41419_2026_9138_MOESM5_ESM.pdf]

1D

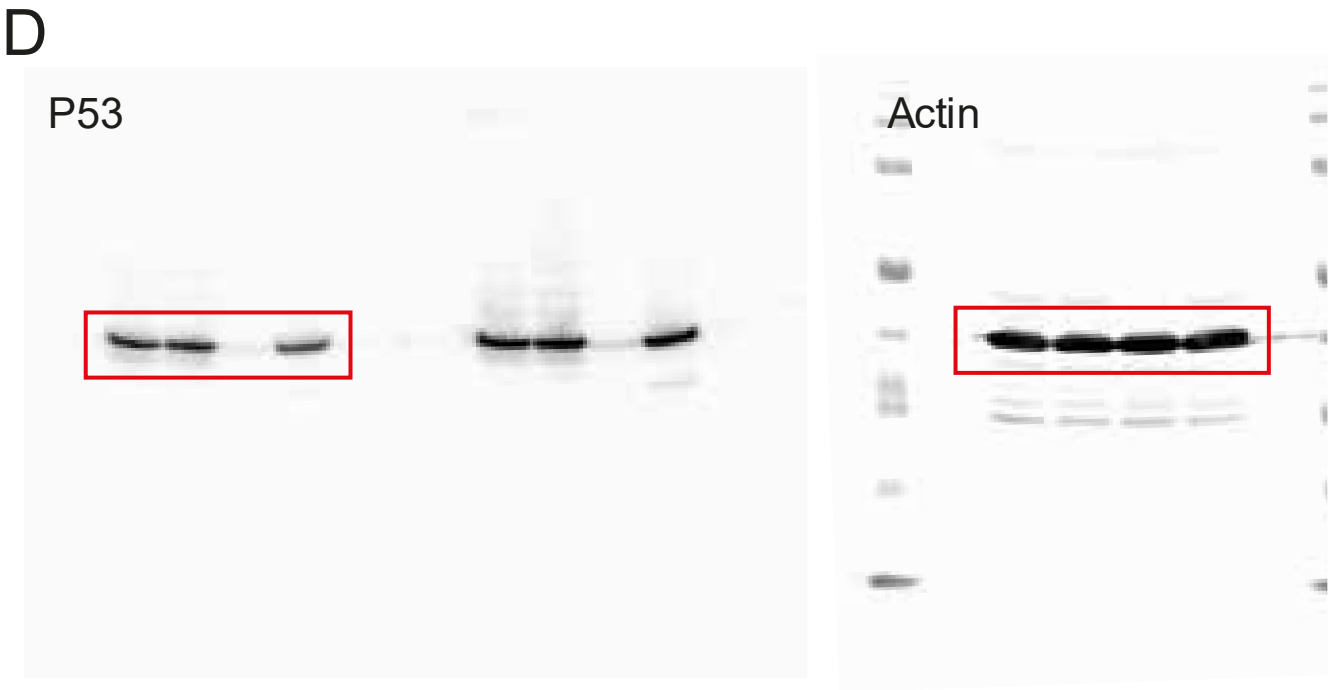

S1A

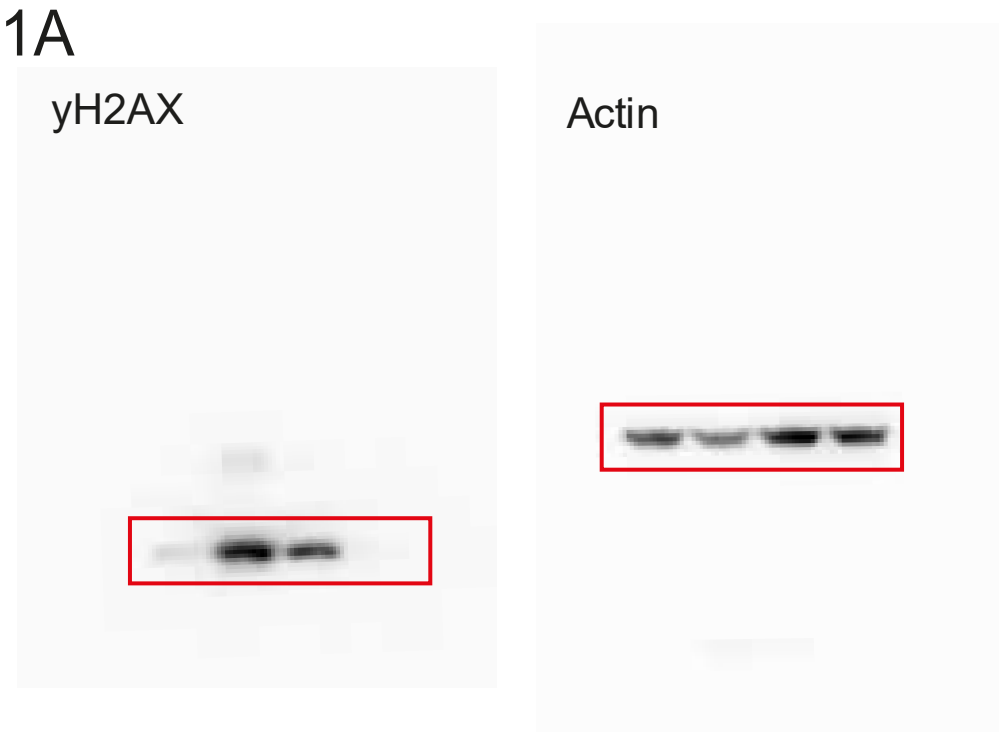

2A

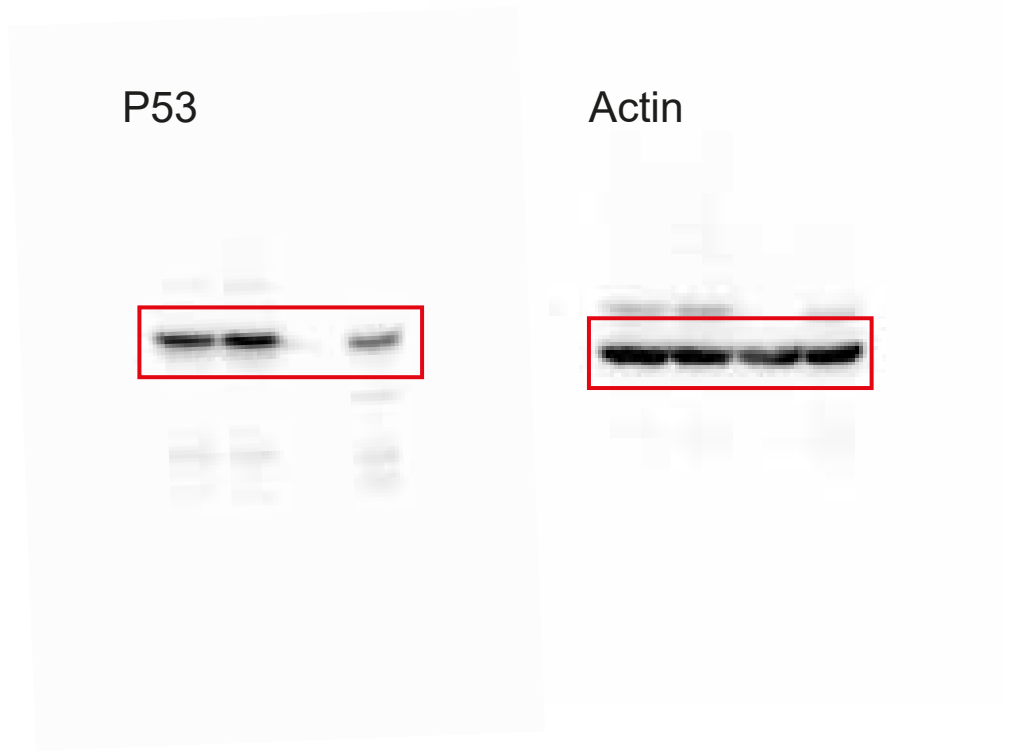

S2B

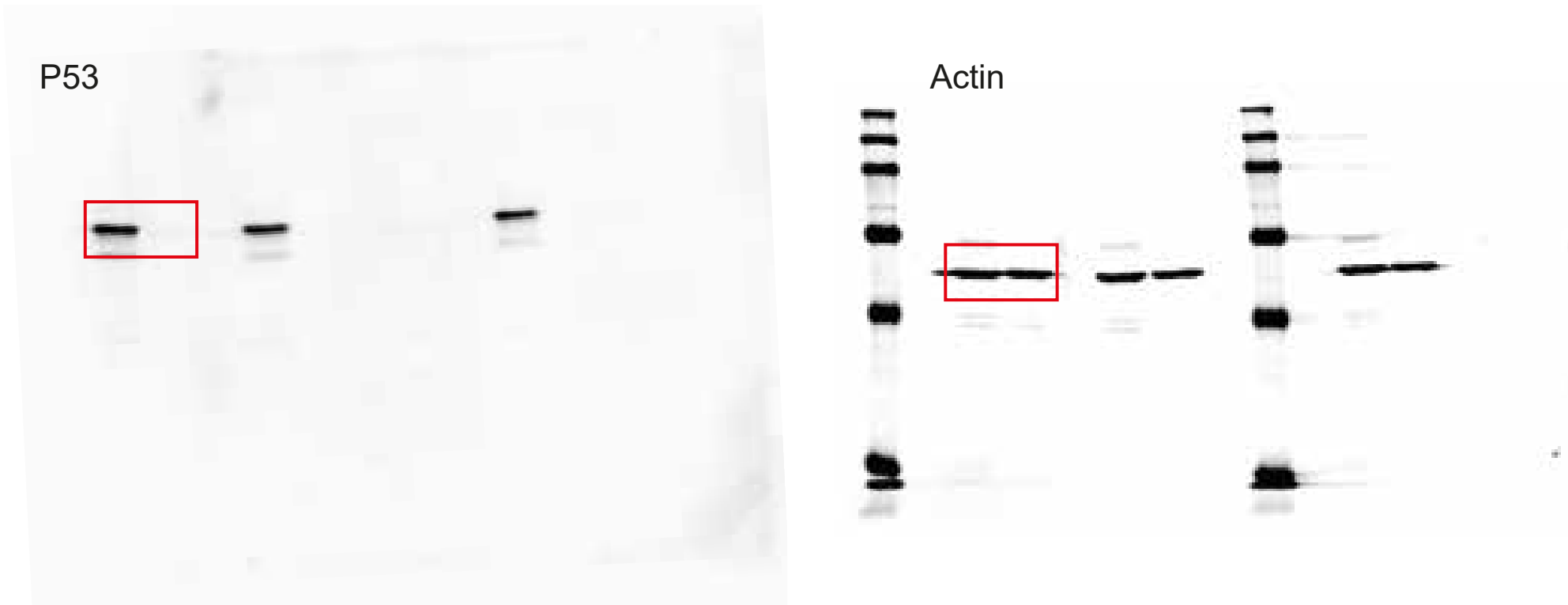

S2D

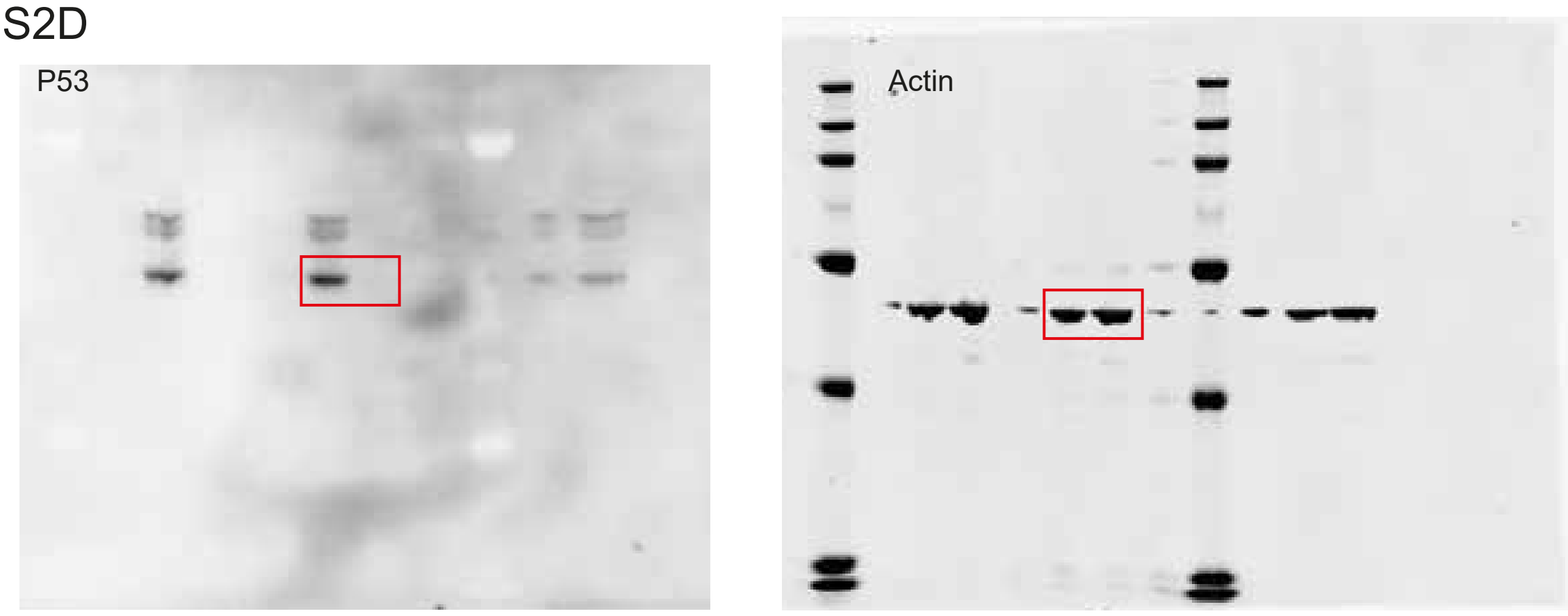

3A

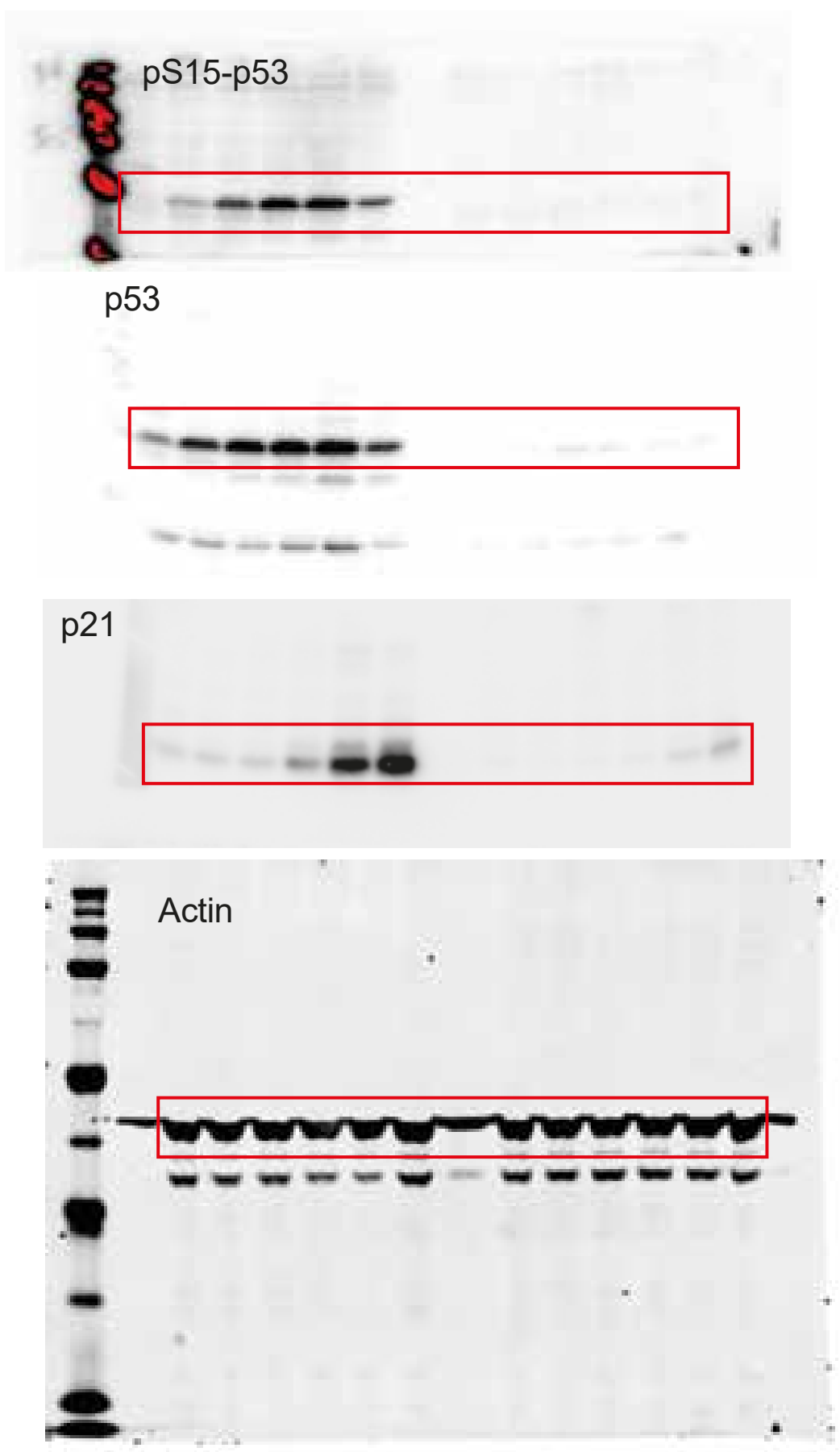

3E

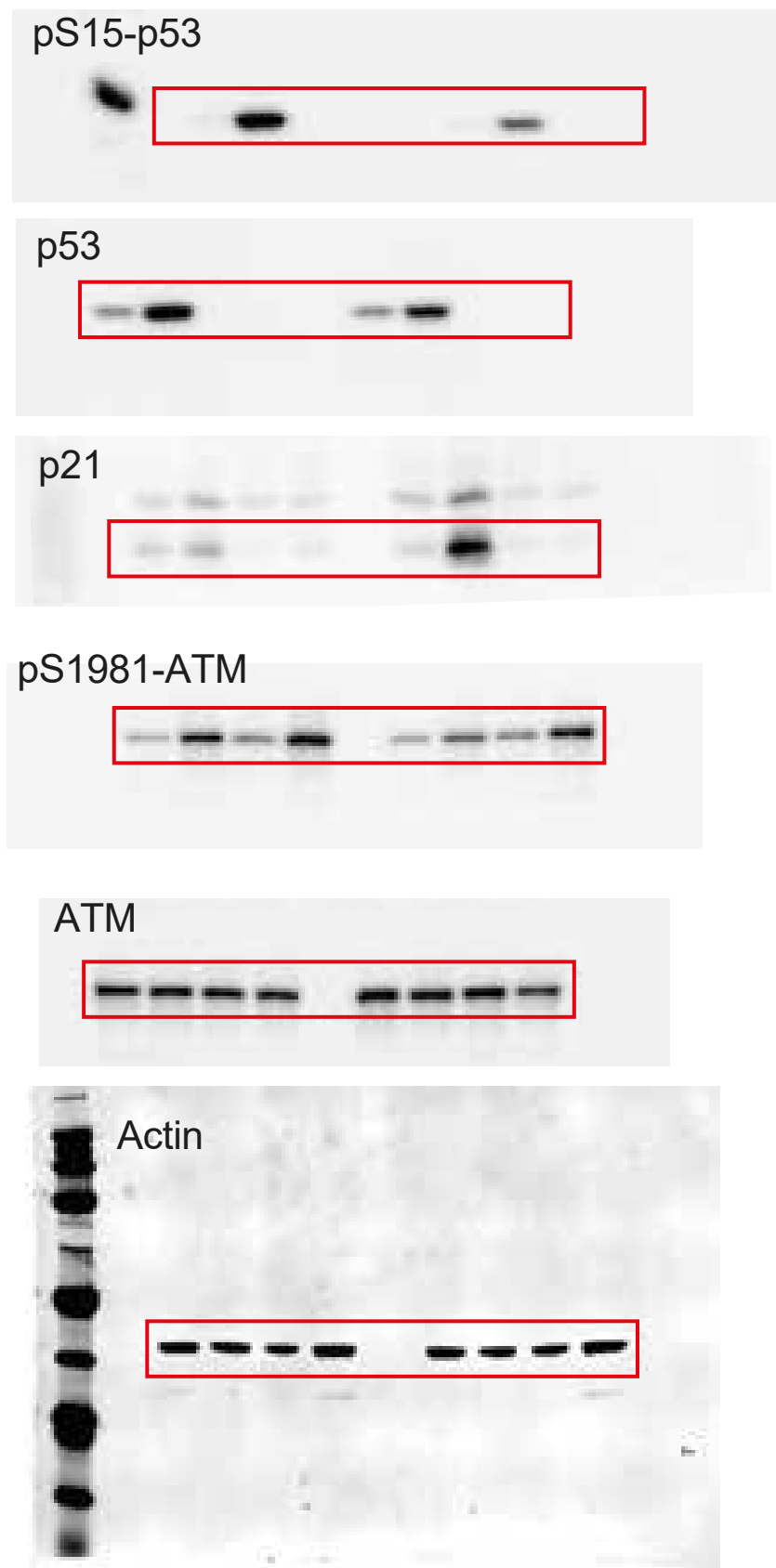

S3F

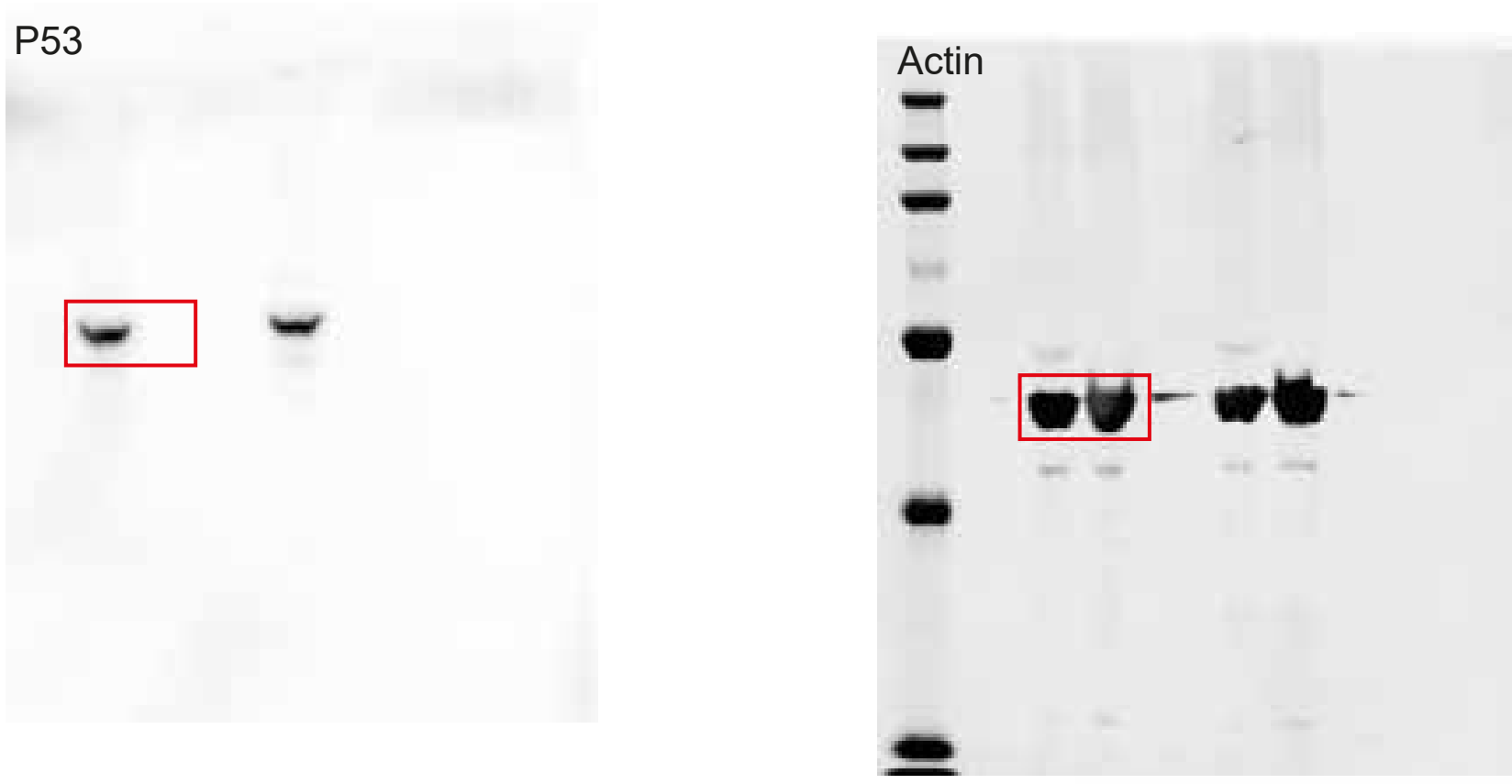

3L

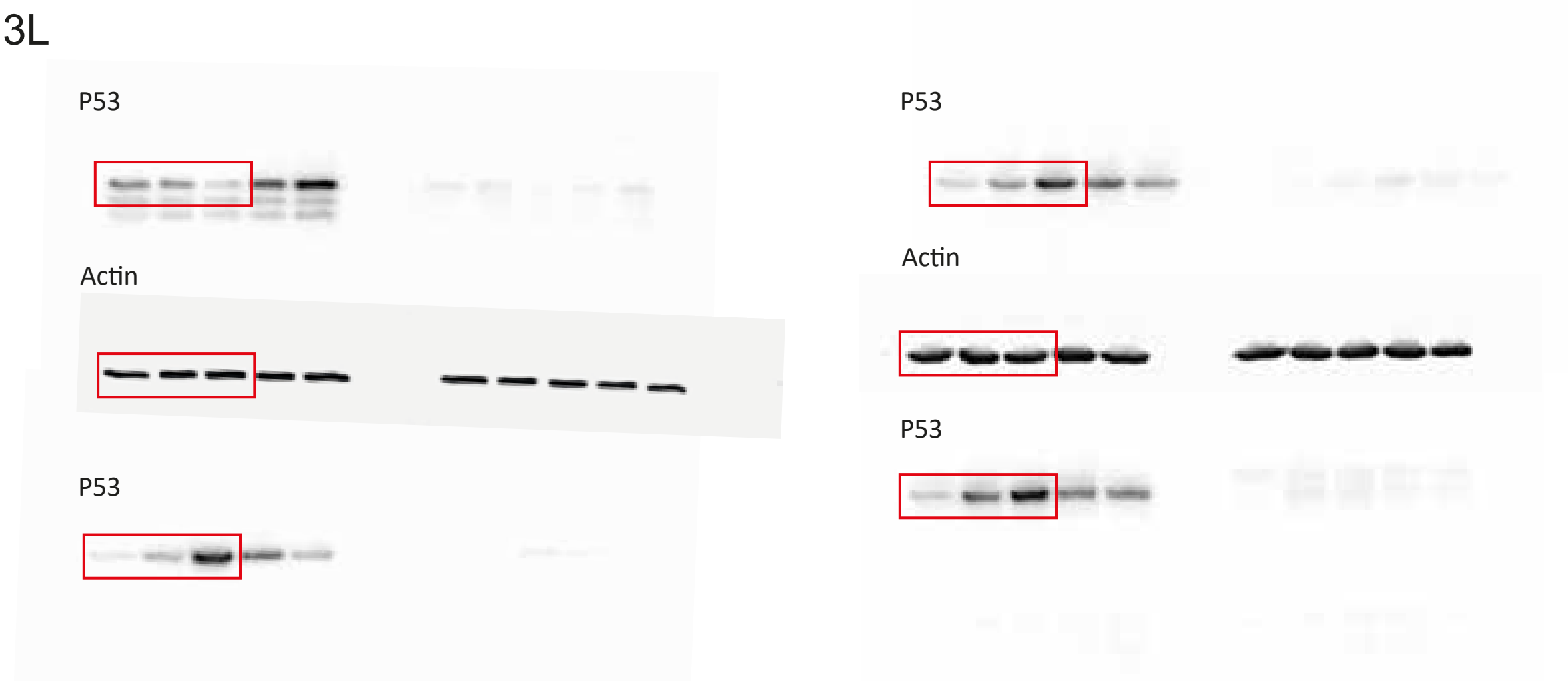

S4A

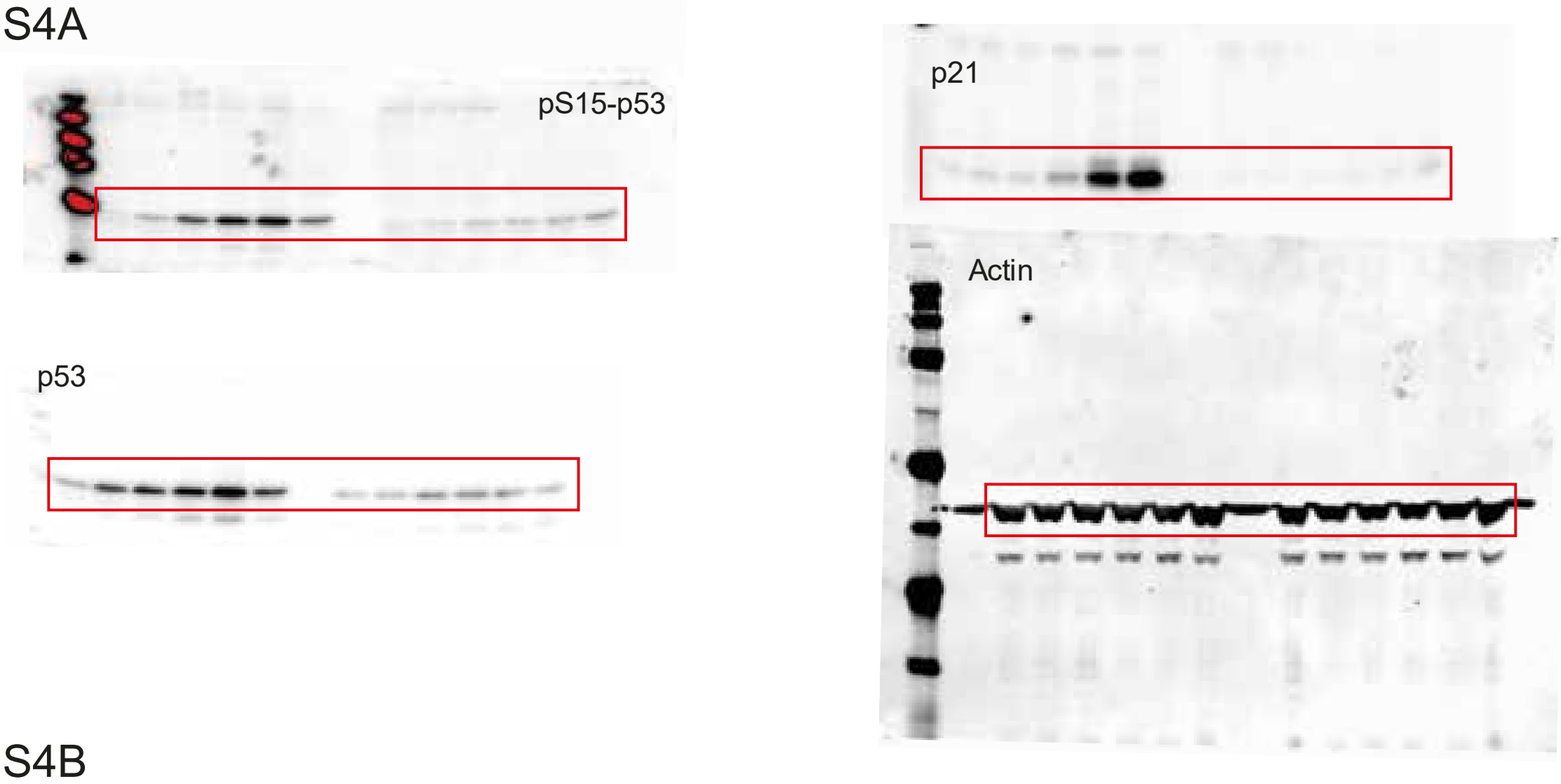

S4B

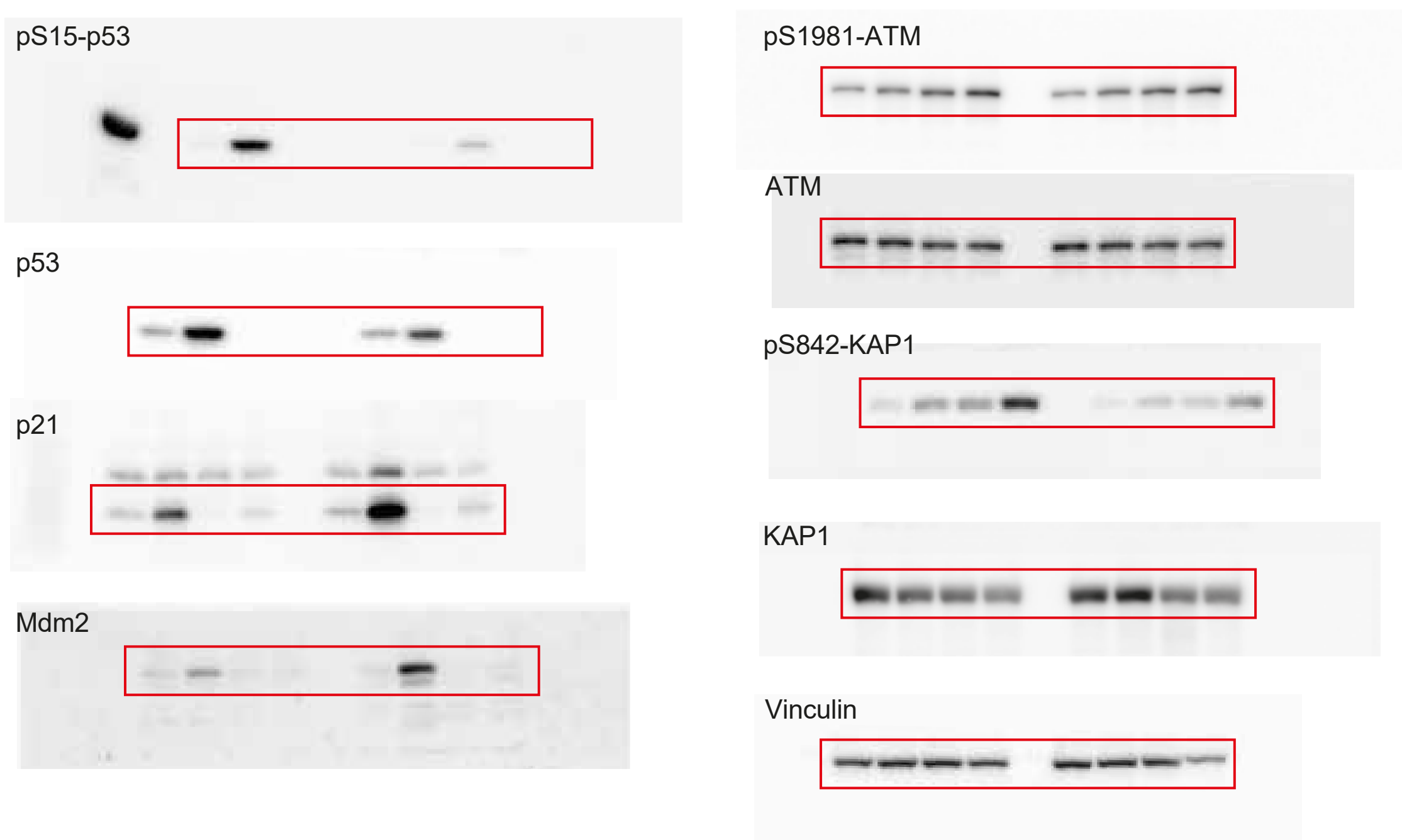

S4D

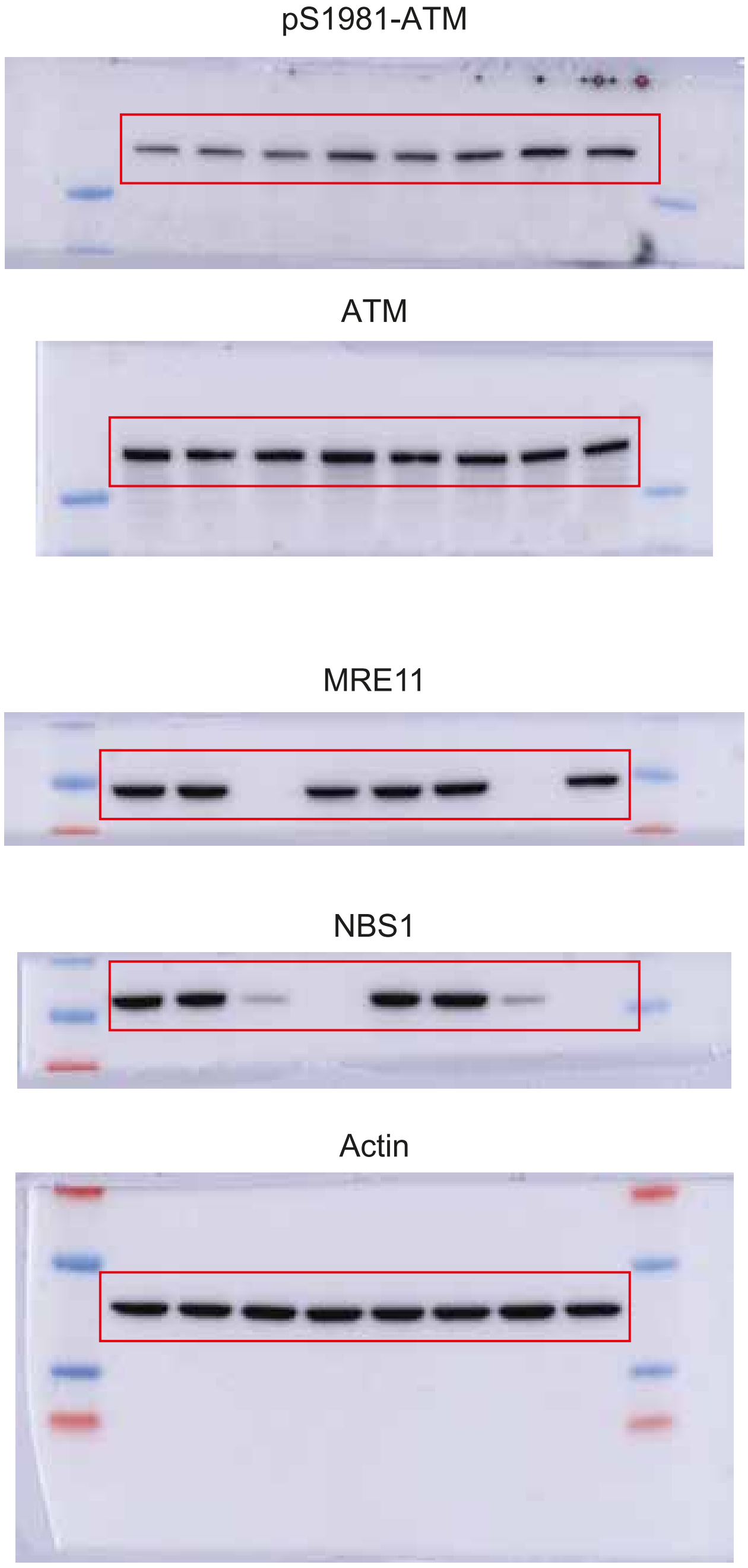
pS1981-ATM
ATM
MRE11
NBS1
Actin

S4E

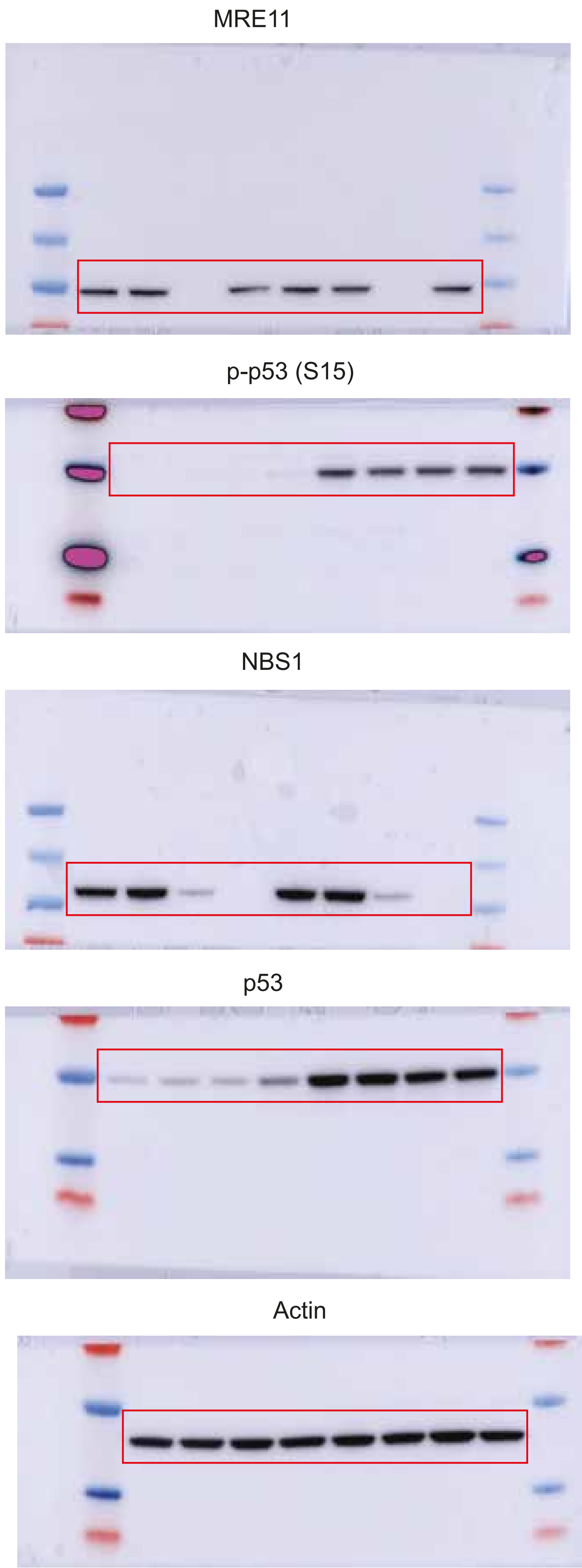
MRE11
p-p53 (S15)
NBS1
p53
Actin

4D

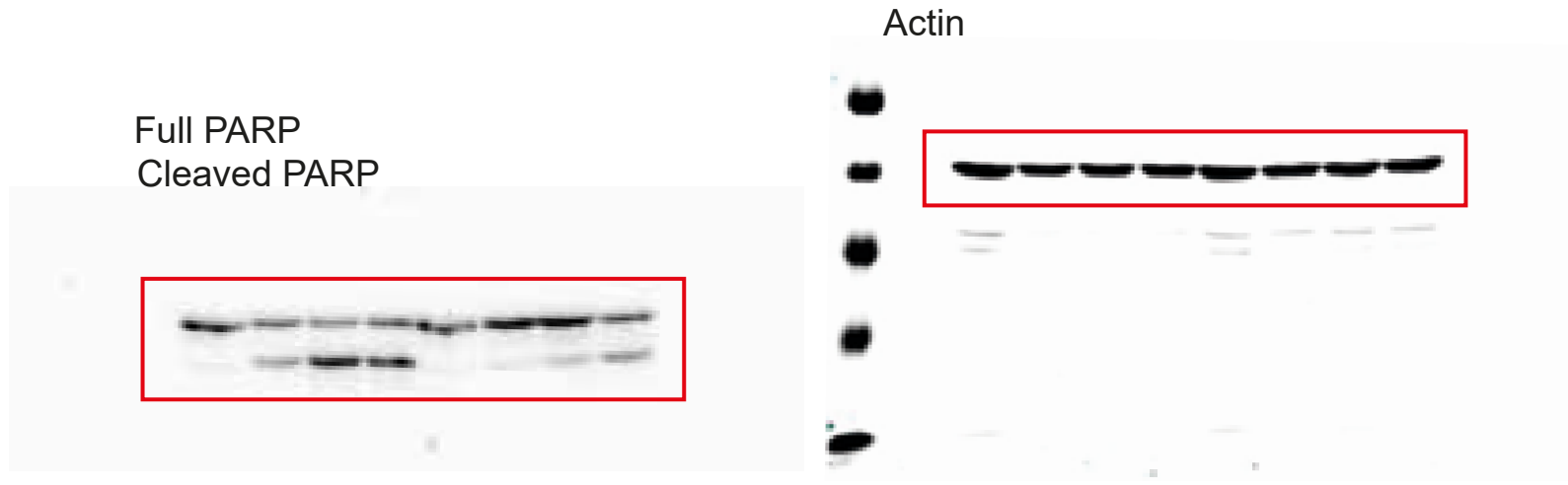
Full PARP
Cleaved PARP
Actin

S6A

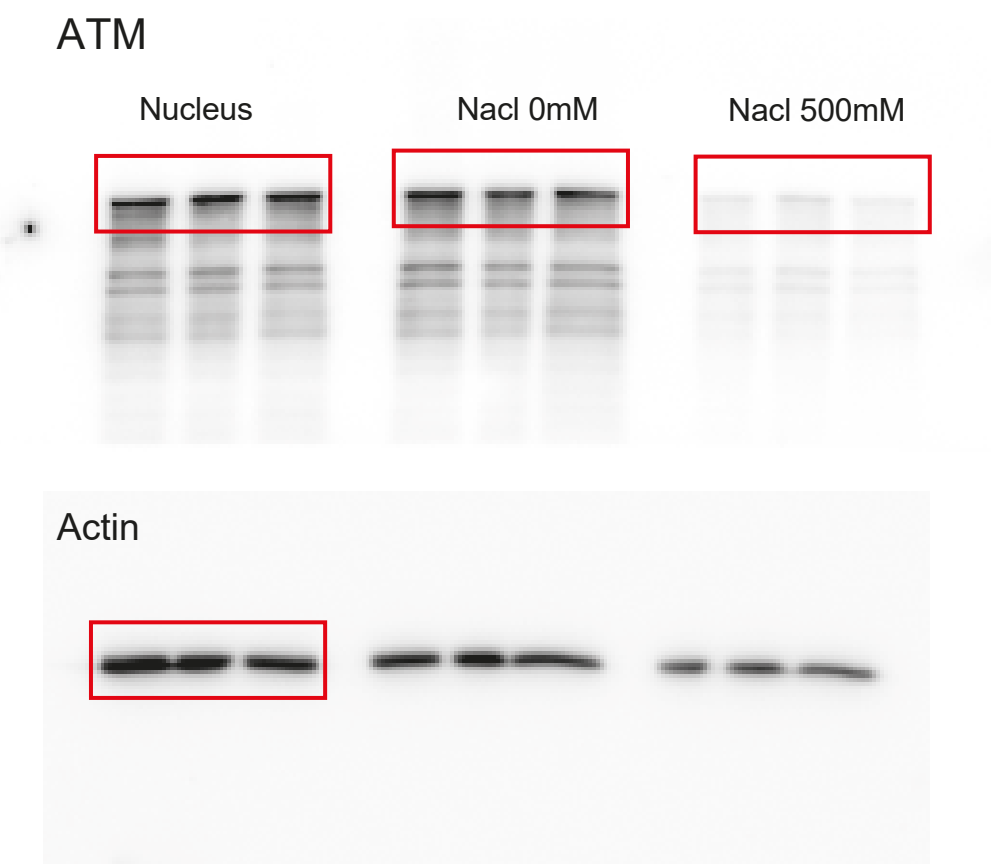
ATM
Nucleus
Nacl 0mM
Nacl 500mM
Actin

S6C

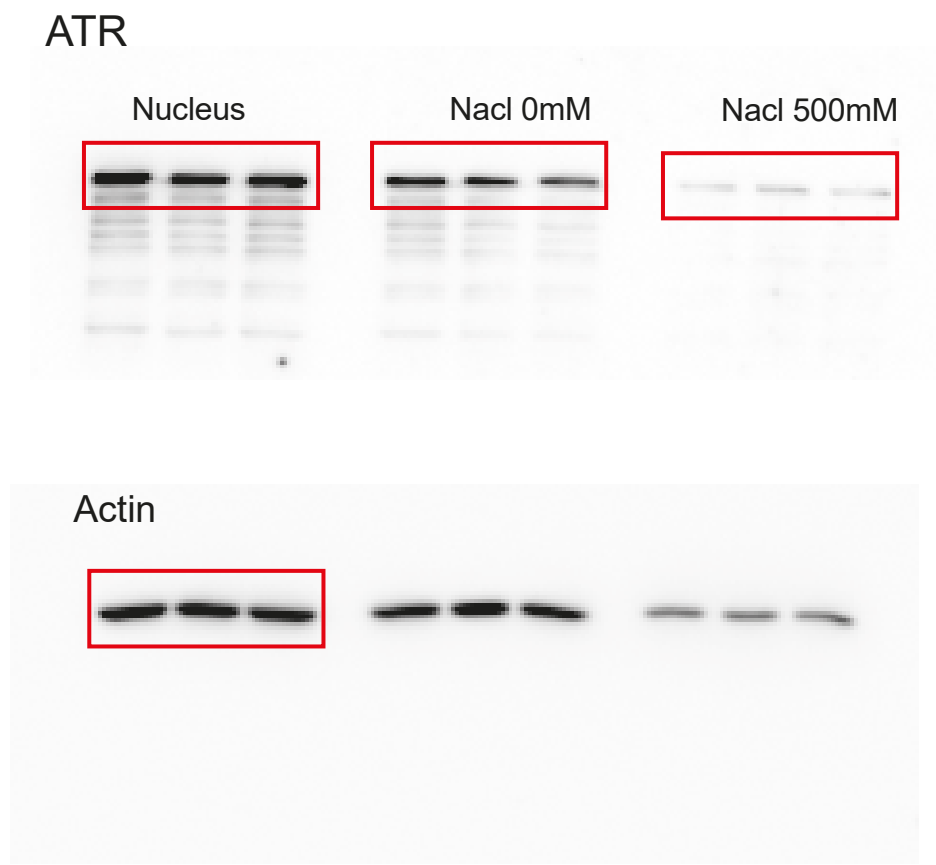
ATR
Nucleus
Nacl 0mM
Nacl 500mM
Actin

5A

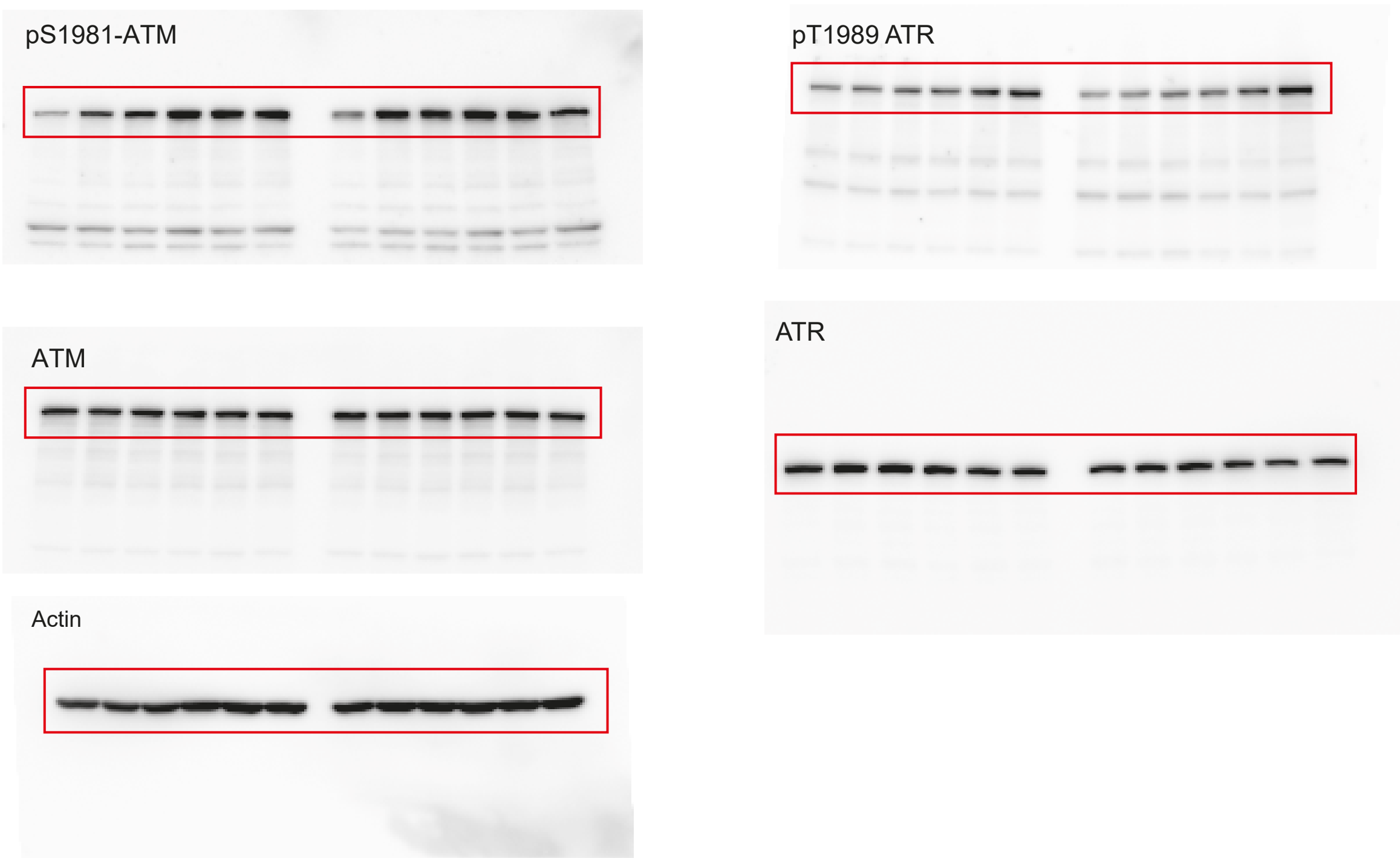
pS1981-ATM
ATM
Actin
pT1989 ATR
ATR

5D

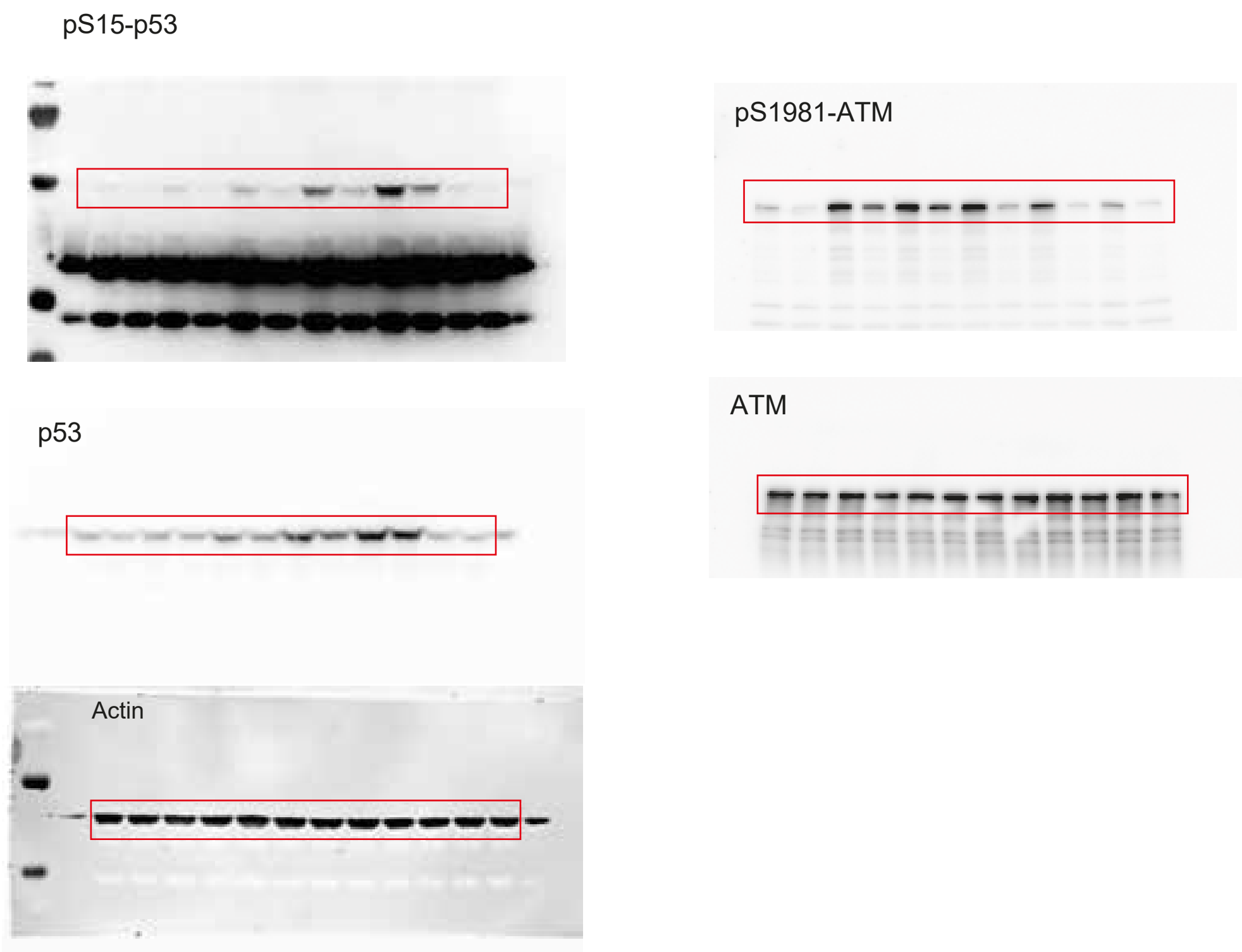
pS15-p53
p53
Actin
pS1981-ATM
ATM

5H

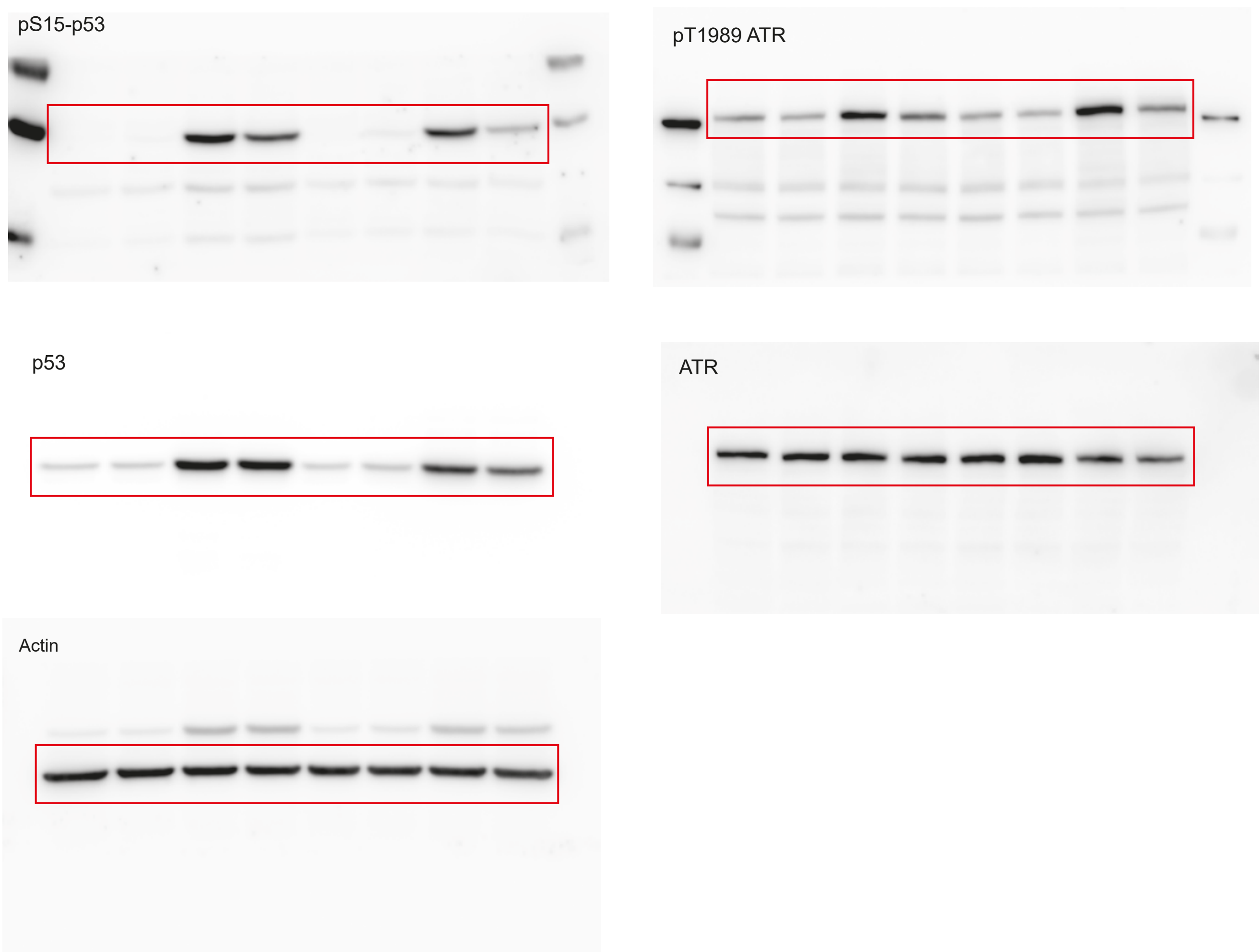
pS15-p53
p53
Actin
pT1989 ATR
ATR
